# Supplementary material for: Influence of hydrometeorological risk factors on child diarrhea and enteropathogens in rural Bangladesh
Source: PLoS Negl Trop Dis. 2024 May 13;18(5):e0012157. doi: 10.1371/journal.pntd.0012157 (PMC11115220; doi:10.1371/journal.pntd.0012157)
Supplement: S1 Text — Includes: A. Deviations from pre-analysis plan. Appendix B. Detection of enteropathogens in stool. Appendix C. Incubation periods for each pathogen outcome. Appendix D. Description of risk factors and data sources. Appendix E. Covariate sets tested for each risk factor. (PDF) [file pntd.0012157.s001.pdf]

Supporting Information for *Influence of hydrometeorological risk factors on child diarrhea and enteropathogens in rural Bangladesh*

Appendix A. Deviations from pre-analysis plan 2
Appendix B. Detection of enteropathogens in stool 4
Appendix C. Incubation periods for each pathogen outcome 5
Appendix D. Description of risk factors and data sources 6
Appendix E. Covariate sets tested for each risk factor 7
References 8

Appendix A. Deviations from pre-analysis plan

1) We pre-specified analyses with a continuous measure of weekly average precipitation, but the distribution was extremely right-skewed. For these reasons, to capture days with heavier rainfall, we created an indicator for whether the weekly sum of precipitation was above or below the median for the study period with 1-, 2-, and 3-week lags. As a sensitivity analysis, we also included a 75th percentile cutoff.

   We pre-specified an analysis to investigate whether there was an interaction between precipitation and an indicator for whether the preceding 60 days had no rainfall, as has been done in prior studies. We were not able to conduct this analysis because above median and heavy rainfall periods were almost never preceded by a dry period within the prior 60 days.

2) We ran analyses for water flow accumulation, the enhanced vegetative index, land use, and population density per our pre-analysis plan. Overall, we did not observe associations with study outcomes. We excluded them from this manuscript. Results are available here: https://osf.io/yt67k/

3) We pre-specified the use of the National Aeronautics and Space Administration (NASA) MODIS MOD11A1 dataset [1] for land surface temperature. However, we found that this dataset had significant missingness for the study location during the rainy season, which we suspect was due to cloud coverage, and we were unable to overcome this with imputation methods. Instead, we used the NASA Famine Early Warning System Network Land Data Assimilation System (FLDAS) Central Asia dataset [2] for near surface air temperature, which had ~0.2% missingness for the study period and location, but had lower spatial granularity.

4) We pre-specified the use of the National Oceanic and Atmospheric Administration/OAR/ESRL Physical Science Laboratory dataset [3,4] for precipitation data. However, given the poor spatial resolution (~55km), we instead used the GloH2O Multiple-Source Weighted-Ensemble Precipitation dataset [5]. This dataset has ~10km spatial resolution on daily precipitation data that incorporates gauge, satellite, and reanalysis data, and corrects for bias.

5) We pre-specified that we would pool across all intervention arms from the original study to maximize statistical power and include subgroup analyses within the control arm or intervention arms (all pooled together). In a parallel analysis, we observed a significant influence of intervention effectiveness on diarrhea in a seasonally-dependent manner, thus we restricted our analysis of the diarrhea outcome to only control arms, or outcomes collected at baseline before interventions were delivered. This abrogated the need to do subgroup analyses for the diarrhea outcome.

6) We pre-specified the use of generalized additive mixed models with a binomial family and logit link for binary outcomes. However, odds ratios obtained from binomial logit models are not a good estimate of the prevalence ratio when the outcome is common, which is the case for many of our pathogen outcomes (e.g. EAEC was detected in 76% of children). Thus, we instead used Poisson regression (log link) for all outcomes, which provides appropriate estimates of the prevalence ratio for all of our outcomes.

Appendix B. Detection of enteropathogens in stool

Real time PCR assays on the TaqMan Array Card and associated gene targets. Assays have been described previously and extensively validated [6,7]. Nucleic acid was extracted with the QIAamp Fast DNA Stool mini kit (Qiagen, Hilden, Germany) with pre-treatment steps that included bead beating. AgPath One Step RT-PCR reagents were used for qPCR reactions, which were performed on ViiA 7 systems. Quantification cycles (C_qs) are the PCR cycle values at which fluorescence from amplification exceeds the background, which acts as an inverse metric of quantity of nucleic acid. Valid results required proper functioning of controls (the negative results of a sample are valid only when its external control MS2 is positive, Cq < 35; the positive results are valid only when the corresponding extraction blank is negative for the relevant targets, Cq > 35), and excluded data flagged by the real time PCR software, i.e., BADROX in combination with NOISE or SPIKE.

Pathogen	Gene
**Virus**	
Adenovirus 40/41	Fiber gene
Astrovirus	Capsid
Norovirus GI/GII	GI ORF1-2 and GII ORF1-2
Rotavirus	*NSP3*
Sapovirus	*RdRp*
**Bacteria**	
Enteroaggregative *Escherichia coli* (EAEC)*	*aaiC*, *aatA*
Enteropathogenic *E. coli* (EPEC)*	*bfpA*, *eae*
Enterotoxigenic *E. coli* (ETEC)*	*LT*, *STh*, *STp*
Shiga toxin-producing *E. coli* (STEC)*	*stx1*, *stx2*
Aeromonas	Aerolysin
Bacteroides fragilis	EGBF
Campylobacter spp.	*cpn60*
Campylobacter jejuni/coli	*cadF*
Clostridium difficile	*tcdA*, *tcdB*
Helicobacter pylori	*ureC*
Plesiomonas shigelloides	*gyrB*
Salmonella enterica	*ttr*
Shigella spp./Enteroinvasive *E. coli* (EIEC)	*ipaH*
Vibrio cholerae	*hlyA*
**Fungi**	
Encephalitozoon intestinalis	SSU rRNA
Enterocytozoon bieneusi	*ITS*
**Protozoa**	
Cryptosporidium spp.	18S rRNA
Entamoeba histolytica	18S rRNA
Entamoeba spp.	18S rRNA
Giardia spp.	18S rRNA
Cyclospora cayetanensis	18S rRNA
Cystoisospora belli	18S rRNA
**Helminth**	
Ancylostoma duodenale	*ITS2*
Ascaris lumbricoides	*ITS1*
Blastocystis spp.	18S rRNA
Hymenolepis nana	*ITS1*
Necator americanus	*ITS2*
Strongyloides stercoralis	Dispersed repetitive sequence
Schistosoma spp.	*ITS*
Trichuris trichiura	18S rRNA
**Controls**	
MS2	*MS2g1*
PhHV	*gB*

* *E. coli* pathotypes were defined as follows: EAEC (*aaiC*, or *aatA*, or both), atypical EPEC (*eae* without *bfpA*, *stx1*, and *stx2*), typical EPEC (*bfpA* and *eae*), ETEC (STh, STp, or LT), STEC (*eae* without *bfpA* and with *stx1*, *stx2*, or both).

Appendix C. Incubation periods for each pathogen outcome

The following incubation periods informed the selection of precipitation lag periods used in Figure 4.

Pathogen	Incubation Period in Literature	Lag Used in Analysis	Reference
Adenovirus 40/41	3-10 days	1 week	[8]
aEPEC [9]	9-12 hours	1 week	[9]
Aeromonas	12-48 hours	1 week	[10]
B. fragilis	1-5 days	1 weeks	[11]
C. difficile	2-3 days	1 week	[12]
Campylobacter	2-3 days	1 week	[13]
Cryptosporidium	2-10 days	2 weeks	[14]
E. bieneusi	5-12 days	2 weeks	[15]
EAEC	8-48 hours	1 week	[9]
Giardia	1-14 days	2 weeks	[16]
LT-ETEC	10-72 hours	1 week	[9]
Norovirus	1.2 days	1 week	[17]
Plesiomonas	48 hours	1 week	[18]
Sapovirus	1.7 days	1 week	[17]
Shigella/EIEC [13]	10-18 hours	1 week	[9,13]
ST-ETEC	10-72 hours	1 week	[9]
STEC	1-10 days	1 week	[9]
tEPEC	9-12 hours	1 week	[9]

Appendix D. Description of risk factors and data sources

Spatial risk factor	Source	Description	Unit of measurement	Temporal resolution	Spatial resolution
Vapor pressure deficit	Terraclimate [19]	The difference between observed water vapor pressure and the water vapor pressure at full air saturation. Low VPD is associated with humid air, while high VPD is associated with dry air.	kPa	Monthly	4 km (1/24th degree)
Surface water in close proximity to household	Global Surface Water Explorer [20]	Multiple variables were used from this dataset to create the following risk factors for analysis:<br>• Proportion of area within radius (250, 500, 750m) with surface water detected:<br>○ Any surface water<br>○ Seasonal surface water<br>○ Ephemeral surface water<br>• Distance tertile from household to:<br>○ Any surface water<br>○ Seasonal surface water<br>○ Ephemeral surface water	months	Monthly	30 meters
Precipitation	GloH2O Multiple-Source Weighted-Ensemble Precipitation dataset [5]	The precipitation accumulation for the geographical area. Used to create variables for:<br>• Average 7-day precipitation, with lags<br>• Heavy rainfall in past week (total precipitation in 24h period within previous 7 days >80th percentile daily precipitation over the study period), with lags<br>• Rainfall categories (based on 33rd and 66th percentile cutoffs over the study period) for the 60 days preceding heavy rainfall measurement, with lags	mm; and also factor: heavy rainfall in past week (Y/N), and previous 60-day rainfall category (high, medium, low).	Daily	55 km (0.5 degree)
Temperature (minimum, maximum, average)	NASA FLDAS Central Asia [2]	A daily near surface air temperature was extracted for each coordinate point throughout the study period. The absolute minimum temperature, absolute maximum temperature, and average temperature were reported for the 7-day and 30-day periods preceding an observation, including lags of 0, 1, 2, and 3 weeks.	°C	Daily	1 km

Appendix E. Covariate sets tested for each risk factor

Risk factor	Potential Confounder
Vapor pressure deficit	Monthly precipitation
Distance from surface water	Number of animals in compound
Frequency of surface water in close proximity to household	Monthly precipitation
Precipitation	None
Temperature	None

In addition to the potential confounders listed above, age, sex, and household wealth quartile were included as potential covariates for all models.

For enteropathogen models, antibiotic use in the previous 7 days, whether the intervention included a water, sanitation, and hygiene (WSH) component, and whether the intervention included a nutrition component were also included as potential covariates.

All covariates were pre-screened with a likelihood ratio test and those associated with the outcome (p-value < 0.1) were included in adjusted models.

If potential spatial autocorrelation was detected after fitting the initial generalized additive mixed model, a bidimensional thin plate spline function of household latitude and longitude was additionally included in the model.

References

1. Wan Z, Hook S, Hulley G. MOD11A1 MODIS/Terra Land Surface Temperature/Emissivity Daily L3 Global 1km SIN Grid V006. NASA EOSDIS Land Processes DAAC; 2015. doi:10.5067/MODIS/MOD11A1.006

2. McNally A, Jacob J, Arsenault K, Slinski K, Sarmiento DP, Hoell A, et al. A Central Asia hydrologic monitoring dataset for food and water security applications in Afghanistan. Earth System Science Data. 2022;14: 3115–3135. doi:10.5194/essd-14-3115-2022

3. Xie P, Chen M, Yang S, Yatagai A, Hayasaka T, Fukushima Y, et al. A Gauge-Based Analysis of Daily Precipitation over East Asia. Journal of Hydrometeorology. 2007;8: 607–626. doi:10.1175/JHM583.1

4. Chen M, Shi W, Xie P, Silva VBS, Kousky VE, Higgins RW, et al. Assessing objective techniques for gauge-based analyses of global daily precipitation. Journal of Geophysical Research: Atmospheres. 2008;113. doi:https://doi.org/10.1029/2007JD009132

5. Beck HE, Wood EF, Pan M, Fisher CK, Miralles DG, Dijk AIJM van, et al. MSWEP V2 Global 3-Hourly 0.1° Precipitation: Methodology and Quantitative Assessment. Bulletin of the American Meteorological Society. 2019;100: 473–500. doi:10.1175/BAMS-D-17-0138.1

6. Liu J, Gratz J, Amour C, Kibiki G, Becker S, Janaki L, et al. A Laboratory-Developed TaqMan Array Card for Simultaneous Detection of 19 Enteropathogens. Journal of Clinical Microbiology. 2013;51: 472–480. doi:10.1128/jcm.02658-12

7. Liu J, Gratz J, Amour C, Nshama R, Walongo T, Maro A, et al. Optimization of quantitative PCR methods for enteropathogen detection. PLoS ONE. 2016;11: 1–11. doi:10.1371/journal.pone.0158199

8. Canada PHA of. Pathogen safety data sheet: Infectious substances – Adenovirus (serotypes 40 and 41). 19 Apr 2011 [cited 3 Aug 2022]. Available: https://www.canada.ca/en/public-health/services/laboratory-biosafety-biosecurity/pathogen-safety-data-sheets-risk-assessment/adenovirus-serotypes-40-41.html

9. Escherichia coli, Diarrheagenic - Chapter 4 - 2020 Yellow Book | Travelers' Health | CDC. [cited 3 Aug 2022]. Available: https://wwwnc.cdc.gov/travel/yellowbook/2020/travel-related-infectious-diseases/escherichia-coli-diarrheagenic

10. Aeromonas hydrophila - an overview | ScienceDirect Topics. [cited 3 Aug 2022]. Available: https://www.sciencedirect.com/topics/agricultural-and-biological-sciences/aeromonas-hydrophila

11. Canada PHA of. Pathogen Safety Data Sheets: Infectious Substances – Bacteroides spp. 19 Apr 2011 [cited 3 Aug 2022]. Available: https://www.canada.ca/en/public-

health/services/laboratory-biosafety-biosecurity/pathogen-safety-data-sheets-risk-assessment/bacteroides.html

12. Vital Signs: Preventing Clostridium difficile Infections. [cited 3 Aug 2022]. Available: https://www.cdc.gov/mmwr/preview/mmwrhtml/mm6109a3.htm

13. Chai SJ, Gu W, O'Connor KA, Richardson LC, Tauxe RV. Incubation periods of enteric illnesses in foodborne outbreaks, United States, 1998–2013. Epidemiol Infect. 2019;147: e285. doi:10.1017/S0950268819001651

14. CDC - DPDx - Cryptosporidiosis. 20 May 2019 [cited 3 Aug 2022]. Available: https://www.cdc.gov/dpdx/cryptosporidiosis/index.html

15. Michlmayr D, de Sousa LA, Müller L, Jokelainen P, Ethelberg S, Vestergaard LS, et al. Incubation period, spore shedding duration, and symptoms of Enterocytozoon bieneusi genotype C infection in a foodborne outbreak in Denmark, 2020. Clin Infect Dis. 2021; ciab949. doi:10.1093/cid/ciab949

16. CDC - DPDx - Giardiasis. 22 Apr 2021 [cited 3 Aug 2022]. Available: https://www.cdc.gov/dpdx/giardiasis/index.html

17. Lee RM, Lessler J, Lee RA, Rudolph KE, Reich NG, Perl TM, et al. Incubation periods of viral gastroenteritis: a systematic review. BMC Infectious Diseases. 2013;13: 446. doi:10.1186/1471-2334-13-446

18. Canada PHA of. Pathogen Safety Data Sheets: Infectious Substances – Plesiomonas shigelloides. 30 Apr 2012 [cited 3 Aug 2022]. Available: https://www.canada.ca/en/public-health/services/laboratory-biosafety-biosecurity/pathogen-safety-data-sheets-risk-assessment/plesiomonas-shigelloides.html

19. Abatzoglou JT, Dobrowski SZ, Parks SA, Hegewisch KC. TerraClimate, a high-resolution global dataset of monthly climate and climatic water balance from 1958–2015. Sci Data. 2018;5: 170191. doi:10.1038/sdata.2017.191

20. Pekel J-F, Cottam A, Gorelick N, Belward AS. High-resolution mapping of global surface water and its long-term changes. Nature. 2016;540: 418–422. doi:10.1038/nature20584
