# Supplementary material for: Influence of hydrometeorological risk factors on child diarrhea and enteropathogens in rural Bangladesh
Source: PLoS Negl Trop Dis. 2024 May 13;18(5):e0012157. doi: 10.1371/journal.pntd.0012157 (PMC11115220; doi:10.1371/journal.pntd.0012157)
Supplement: S1 Fig — Bivariate scatter plot of continuous climatic risk factors in the diarrhea cohort. Correlation ellipses depict the strength of the association on the basis of the Spearman rank correlation, color of the ellipse indicates the direction of the correlation, and the correlation coefficient is printed inside each ellipse. (PDF) [file pntd.0012157.s002.pdf]

S1 Figure. Pairwise relationships between weather variables

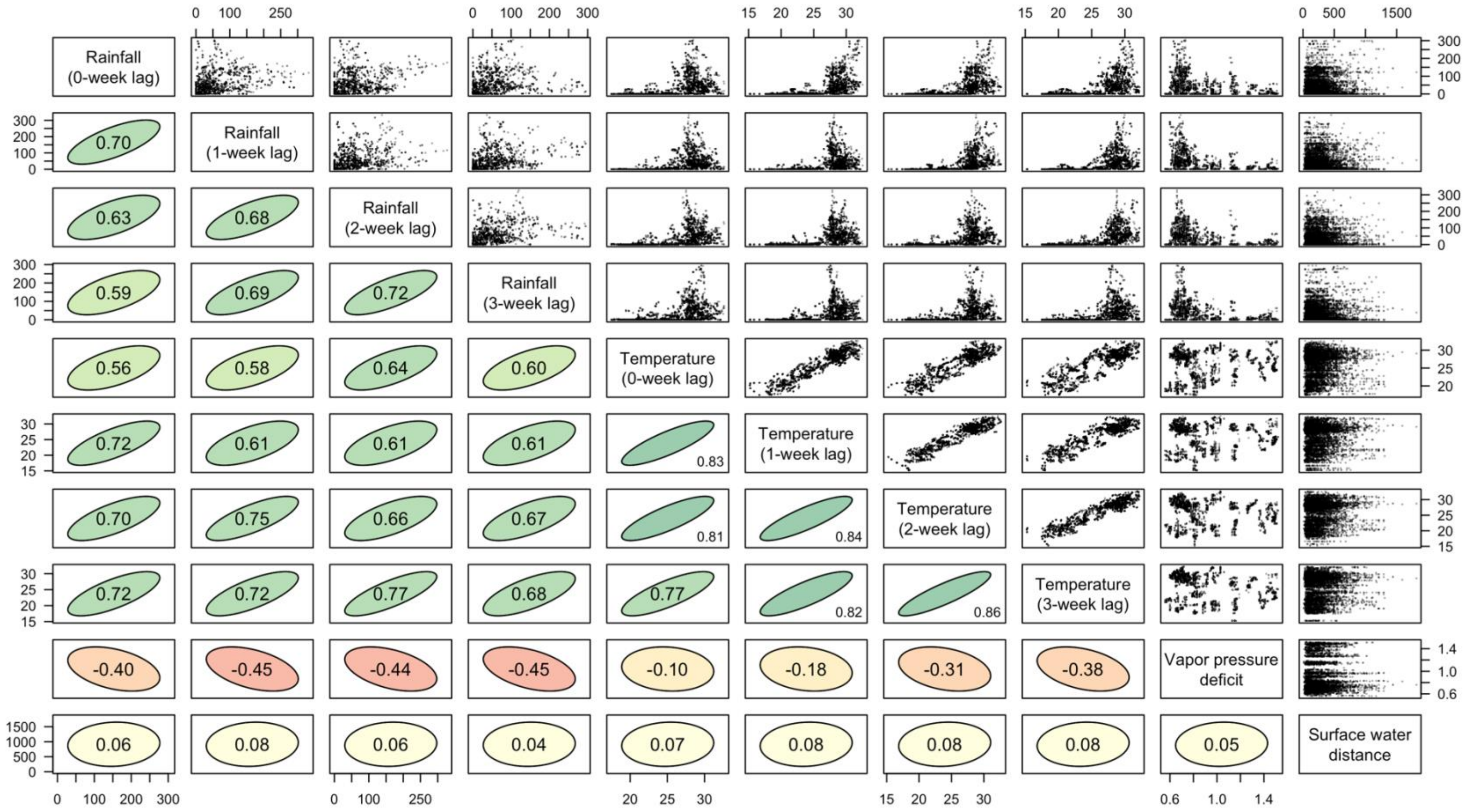

Bivariate scatter plot of continuous climatic risk factors in the diarrhea cohort. Correlation ellipses depict the strength of the association on the basis of the Spearman rank correlation, color of the ellipse indicates the direction of the correlation, and the correlation coefficient is printed inside each ellipse.
