# Supplementary material for: Influence of hydrometeorological risk factors on child diarrhea and enteropathogens in rural Bangladesh
Source: PLoS Negl Trop Dis. 2024 May 13;18(5):e0012157. doi: 10.1371/journal.pntd.0012157 (PMC11115220; doi:10.1371/journal.pntd.0012157)
Supplement: S3 Fig — All panels present adjusted models for temperature with a 1-week lag period for diarrhea measurements in children aged 6 months—5.5 years in the control arms in the original trial. Shaded bands indicate simultaneous 95% confidence intervals accounting for clustering. Prevalence estimates were predicted under conditions which held all adjustment covariates at fixed representative values (see Methods for details). (PDF) [file pntd.0012157.s004.pdf]

S3 Figure. Predicted diarrhea prevalence by temperature minimum, mean, and maximum

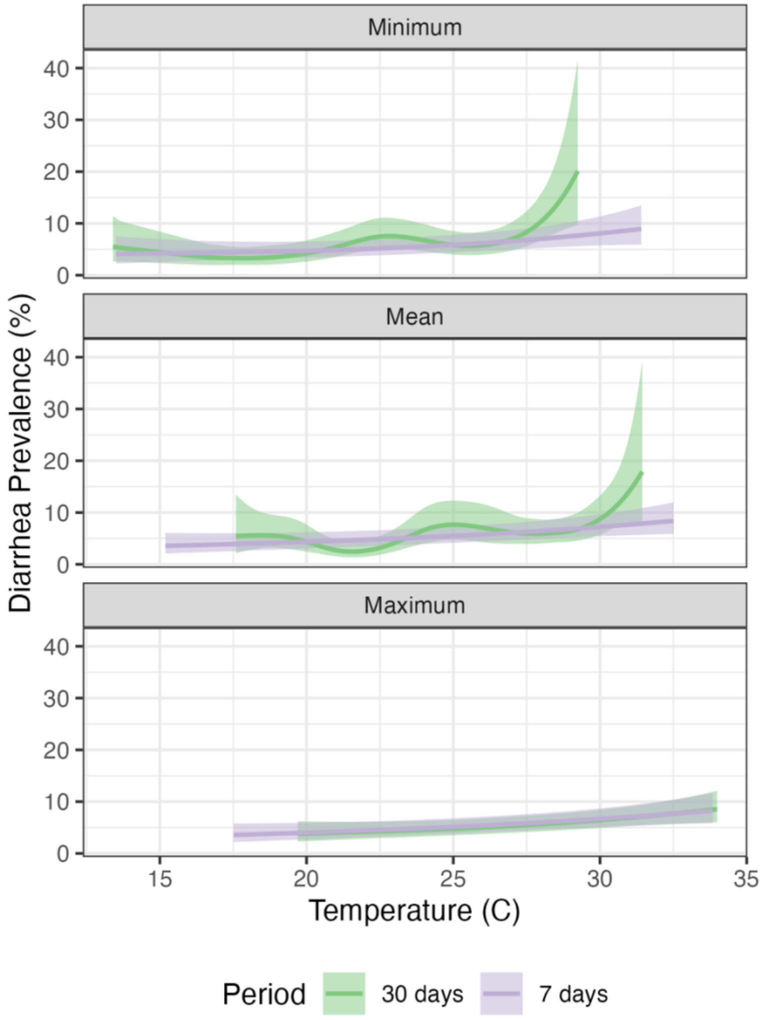

All panels present adjusted models for temperature with a 1-week lag period for diarrhea measurements in children aged 6 months - 5.5 years in the control arms in the original trial. Shaded bands indicate simultaneous 95% confidence intervals accounting for clustering. Prevalence estimates were predicted under conditions which held all adjustment covariates at fixed representative values (see Methods for details).
