# Supplementary material for: Influence of hydrometeorological risk factors on child diarrhea and enteropathogens in rural Bangladesh
Source: PLoS Negl Trop Dis. 2024 May 13;18(5):e0012157. doi: 10.1371/journal.pntd.0012157 (PMC11115220; doi:10.1371/journal.pntd.0012157)
Supplement: S4 Fig — All panels present adjusted models for children approximately 14 months of age in the control, combined water + sanitation + handwashing (WASH), nutrition, and combined nutrition + WASH arms of the original trial. Shaded bands indicate simultaneous 95% confidence intervals accounting for clustering. Prevalence estimates were predicted under conditions which held all adjustment covariates at fixed representative values (see Methods for details). (PDF) [file pntd.0012157.s005.pdf]

S4 Figure. Predicted enteropathogen prevalence by temperature minimum

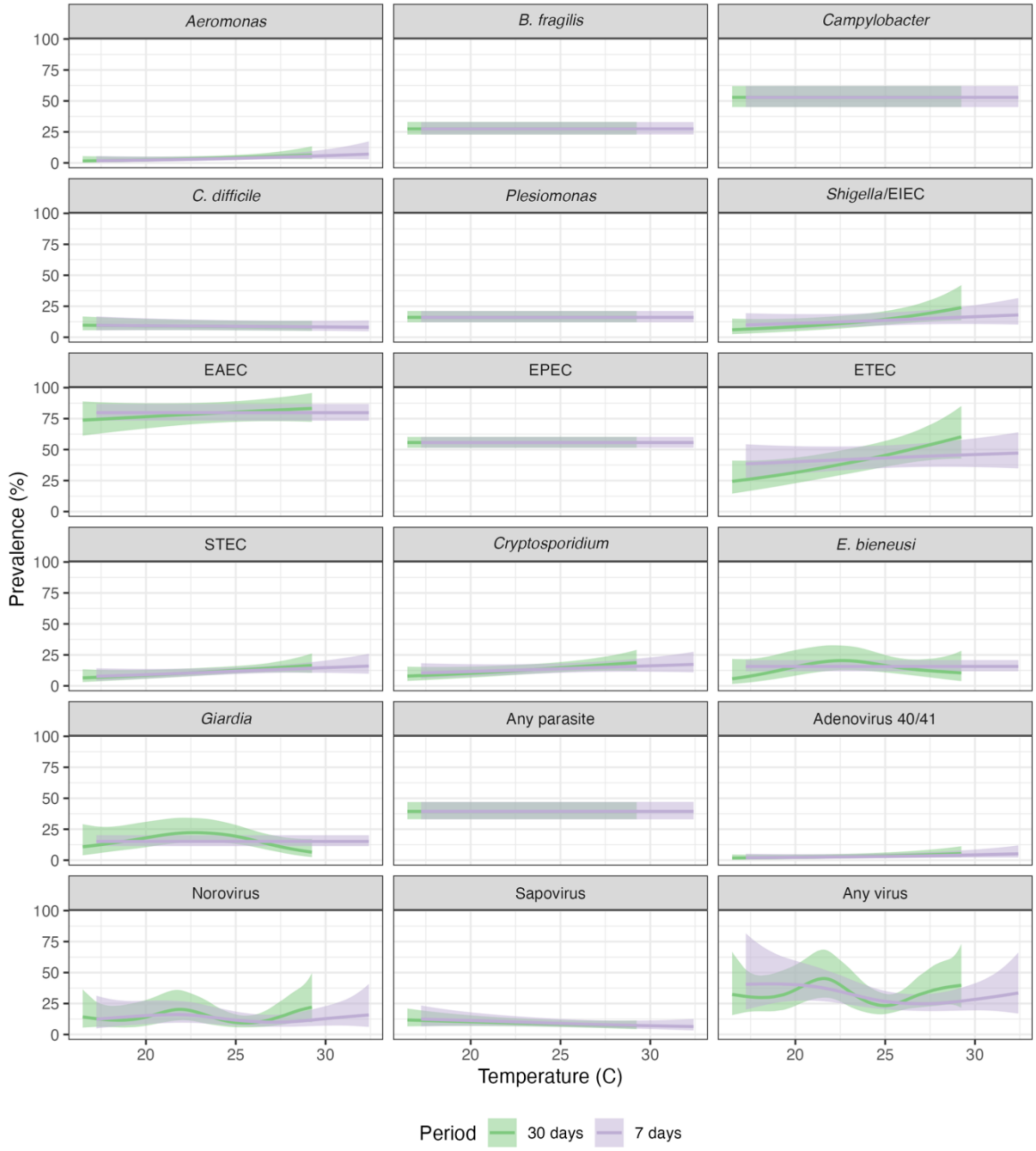

All panels present adjusted models for children approximately 14 months of age in the control, combined water + sanitation + handwashing (WASH), nutrition, and combined nutrition + WASH arms of the original trial. Shaded bands indicate simultaneous 95% confidence intervals accounting for clustering. Prevalence estimates were predicted under conditions which held all adjustment covariates at fixed representative values (see Methods for details).
