# Supplementary material for: Influence of hydrometeorological risk factors on child diarrhea and enteropathogens in rural Bangladesh
Source: PLoS Negl Trop Dis. 2024 May 13;18(5):e0012157. doi: 10.1371/journal.pntd.0012157 (PMC11115220; doi:10.1371/journal.pntd.0012157)
Supplement: S6 Fig — All panels present adjusted models including an indicator for above 75th (58mm) or 90th (105mm) percentile average weekly precipitation as the independent variable; unadjusted models produced similar results. Error bars present 95% confidence intervals adjusted for clustering. The x-axis is on the log scale. Panel A) includes diarrhea measurements in children aged 6 months—5.5 years in the control arms in the original trial. Panels B-D) include measurements in children approximately 14 months of age in the control, combined water + sanitation + handwashing (WASH), nutrition, and combined nutrition + WASH arms of the original trial. Closed circles in panels B-D indicate the expected most important lag based on enteropathogen-specific incubation times (Appendix C in S1 Text). (PDF) [file pntd.0012157.s007.pdf]

S6 Figure. Diarrhea and enteropathogen prevalence by above- vs. below-cutoff for total weekly precipitation at 75th and 90th percentile cutoffs

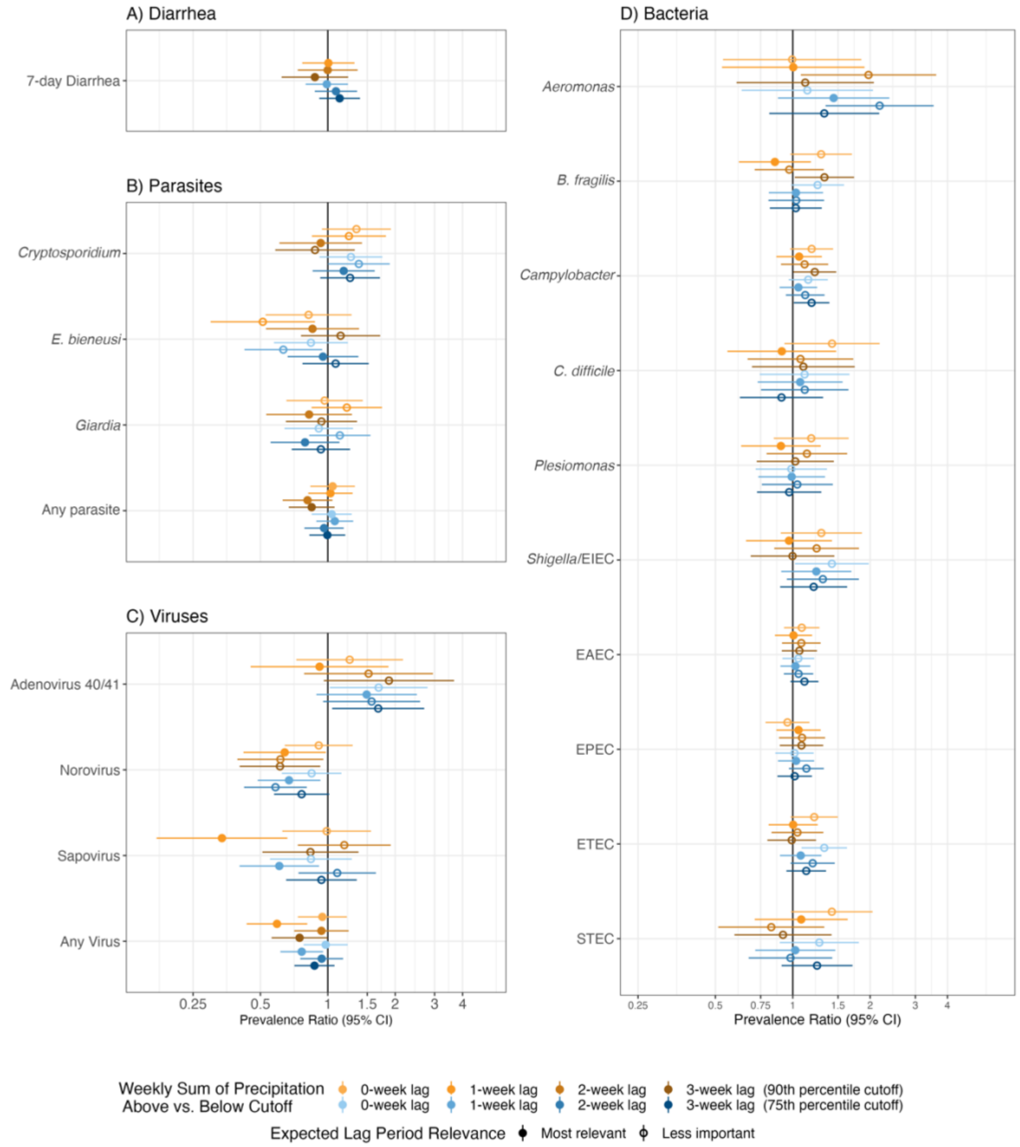

All panels present adjusted models including an indicator for above 75th (58mm) or 90th (105mm) percentile average weekly precipitation as the independent variable; unadjusted models produced similar results. Error bars present 95% confidence intervals adjusted for clustering. The x-axis is on the log scale. Panel A) includes diarrhea measurements in children aged 6 months - 5.5 years in the control arms in the original trial. Panels B-D) include measurements in children approximately 14 months of age in the control, combined water + sanitation + handwashing (WASH), nutrition, and combined nutrition + WASH arms of the original trial. Closed circles in panels B-D indicate the expected most important lag based on enteropathogen-specific incubation times (Appendix C in S1 Text).
