# Supplementary material for: Influence of hydrometeorological risk factors on child diarrhea and enteropathogens in rural Bangladesh
Source: PLoS Negl Trop Dis. 2024 May 13;18(5):e0012157. doi: 10.1371/journal.pntd.0012157 (PMC11115220; doi:10.1371/journal.pntd.0012157)
Supplement: S7 Fig — All panels present adjusted models including an indicator variable for heavy rainfall (total weekly precipitation > 80th (17mm) or 90th (29mm) percentile during the study period); unadjusted models produced similar results. Error bars present 95% confidence intervals adjusted for clustering. The x-axis is on the log scale. Panel A) includes diarrhea measurements in children aged 6 months—5.5 years in the control arms in the original trial. Panels B-D) include measurements in children approximately 14 months of age in the control, combined water + sanitation + handwashing (WASH), nutrition, and combined nutrition + WASH arms of the original trial. Closed circles in panels B-D indicate the expected most important lag based on enteropathogen-specific incubation times (Appendix C in S1 Text). (PDF) [file pntd.0012157.s008.pdf]

S7 Figure. Diarrhea and enteropathogen prevalence by heavy rainfall

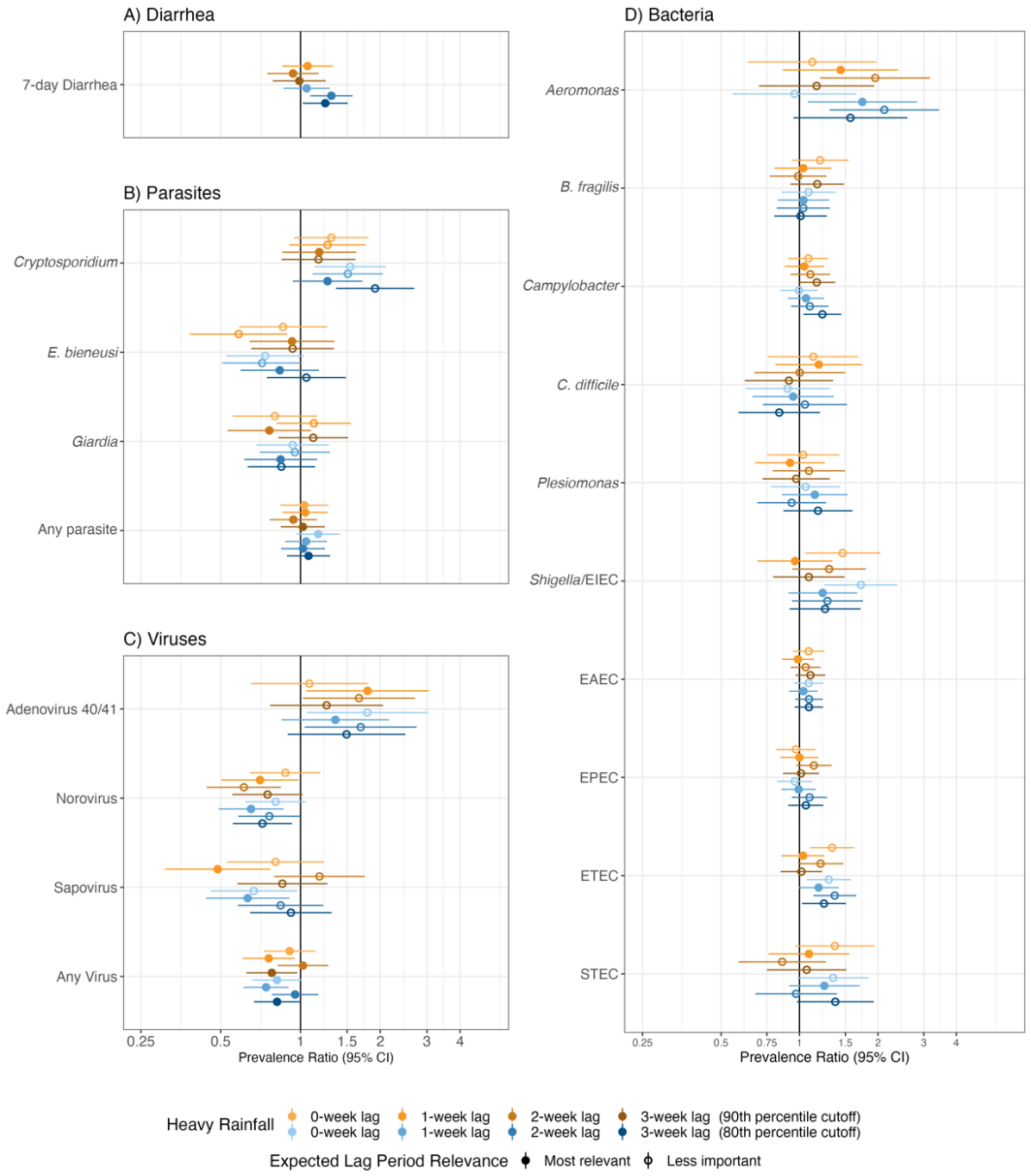

All panels present adjusted models including an indicator variable for heavy rainfall (total weekly precipitation > 80th (17mm) or 90th (29mm) percentile during the study period); unadjusted models produced similar results. Error bars present 95% confidence intervals adjusted for clustering. The x-axis is on the log scale. Panel A) includes diarrhea measurements in children aged 6 months - 5.5 years in the control arms in the original trial. Panels B-D) include measurements in children approximately 14 months of age in the control, combined water + sanitation + handwashing (WASH), nutrition, and combined nutrition + WASH arms of the original trial. Closed circles in panels B-D indicate the expected most important lag based on enteropathogen-specific incubation times (Appendix C in S1 Text).
