# Supplementary material for: Influence of hydrometeorological risk factors on child diarrhea and enteropathogens in rural Bangladesh
Source: PLoS Negl Trop Dis. 2024 May 13;18(5):e0012157. doi: 10.1371/journal.pntd.0012157 (PMC11115220; doi:10.1371/journal.pntd.0012157)
Supplement: S8 Fig — All panels present adjusted models; unadjusted models produced similar results. The independent variable was a categorical variable for tertiles of distance from each household to the nearest surface water (<165m; 165m to <316m, 3316m). Error bars present 95% confidence intervals adjusted for clustering. The x-axis is on the log scale. Panel A) includes diarrhea measurements in children aged 6 months—5.5 years in the control arms in the original trial. Panels B-D) include measurements in children approximately 14 months of age in the control, combined water + sanitation + handwashing (WASH), nutrition, and combined nutrition + WASH arms of the original trial. (PDF) [file pntd.0012157.s009.pdf]

Supporting Information for *Influence of hydrometeorological risk factors on child diarrhea and enteropathogens in rural Bangladesh*

S8 Figure. Prevalence ratios for diarrhea and enteropathogen carriage and distance from study households to surface water

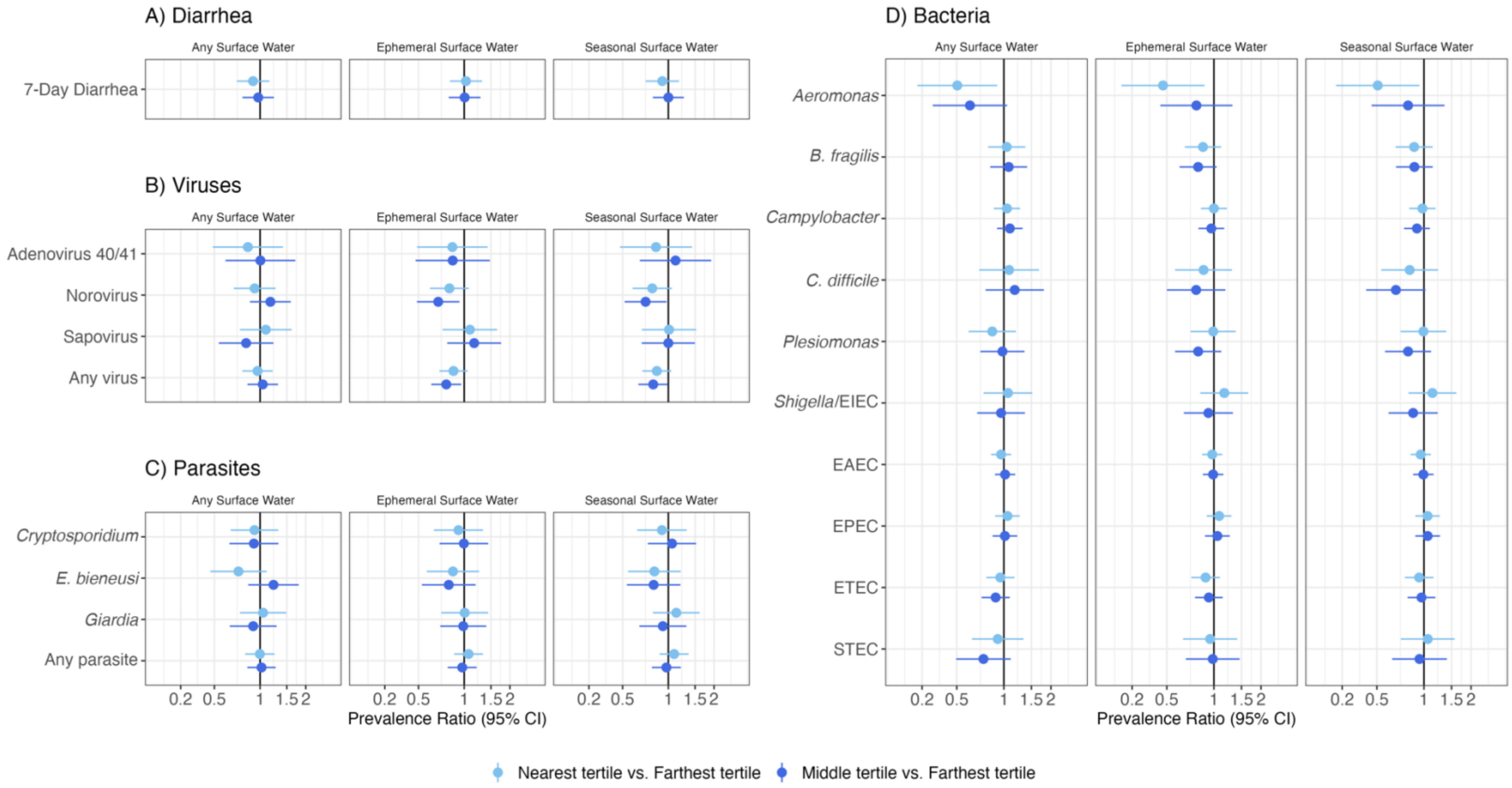

All panels present adjusted models; unadjusted models produced similar results. The independent variable was a categorical variable for tertiles of distance from each household to the nearest surface water (<165m; 165m to <316m, ≥316m). Error bars present 95% confidence intervals adjusted for clustering. The x-axis is on the log scale. Panel A) includes diarrhea measurements in children aged 6 months - 5.5 years in the control arms in the original trial. Panels B-D) include measurements in children approximately 14 months of age in the control, combined water + sanitation + handwashing (WASH), nutrition, and combined nutrition + WASH arms of the original trial.
