# Supplementary material for: Influence of hydrometeorological risk factors on child diarrhea and enteropathogens in rural Bangladesh
Source: PLoS Negl Trop Dis. 2024 May 13;18(5):e0012157. doi: 10.1371/journal.pntd.0012157 (PMC11115220; doi:10.1371/journal.pntd.0012157)
Supplement: S9 Fig — All panels present adjusted models; unadjusted models produced similar results. The independent variable was an indicator for whether the proportion of pixels with surface water within 250m, 500m, 750m of each household was above or below the median. Error bars present 95% confidence intervals adjusted for clustering. The x-axis is on the log scale. Panel A) includes diarrhea measurements in children aged 6 months—5.5 years in the control arms in the original trial. Panels B-D) include measurements in children approximately 14 months of age in the control, combined water + sanitation + handwashing (WASH), nutrition, and combined nutrition + WASH arms of the original trial. (PDF) [file pntd.0012157.s010.pdf]

Supporting Information for *Influence of hydrometeorological risk factors on child diarrhea and enteropathogens in rural Bangladesh*

S9 Figure. Prevalence ratios for diarrhea and enteropathogen carriage associated with the proportion of land around study households that contained surface water

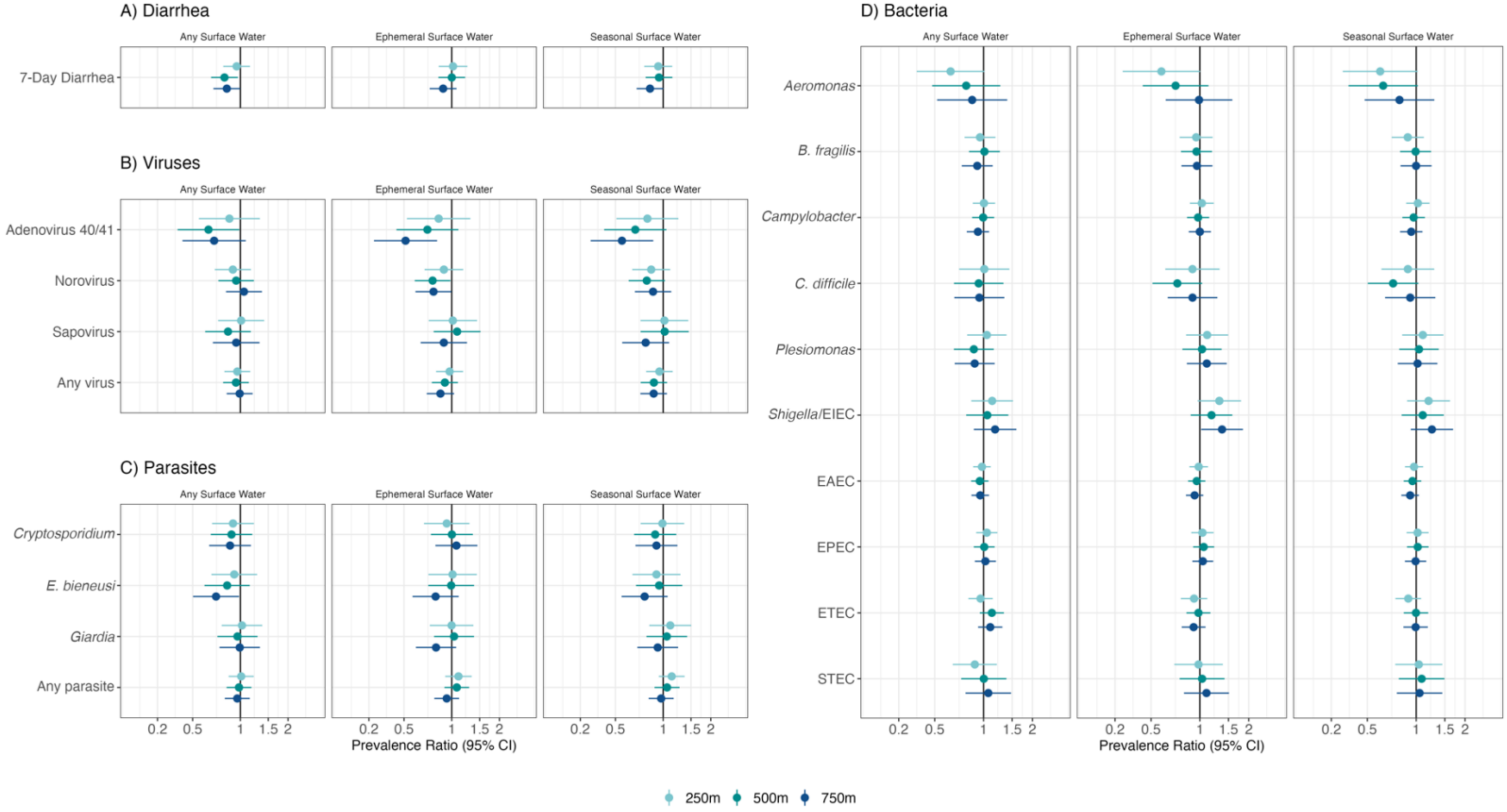

All panels present adjusted models; unadjusted models produced similar results. The independent variable was an indicator for whether the proportion of pixels with surface water within 250m, 500m, 750m of each household was above or below the median. Error bars present 95% confidence intervals adjusted for clustering. The x-axis is on the log scale. Panel A) includes diarrhea measurements in children aged 6 months - 5.5 years in the control arms in the original trial. Panels B-D) include measurements in children approximately 14 months of age in the control, combined water + sanitation + handwashing (WASH), nutrition, and combined nutrition + WASH arms of the original trial.
