# Supplementary material for: Influence of hydrometeorological risk factors on child diarrhea and enteropathogens in rural Bangladesh
Source: PLoS Negl Trop Dis. 2024 May 13;18(5):e0012157. doi: 10.1371/journal.pntd.0012157 (PMC11115220; doi:10.1371/journal.pntd.0012157)
Supplement: S10 Fig — All panels present adjusted models; shaded bands indicate simultaneous 95% confidence intervals accounting for clustering. Pathogen models included measurements in children approximately 14 months of age in the control, combined water + sanitation + handwashing (WASH), nutrition, and combined nutrition + WASH arms of the original trial. Diarrhea model includes measurements in children aged 6 months—5.5 years in the control arms in the original trial. Prevalence estimates were predicted under conditions which held all adjustment covariates at fixed representative values (see Methods for details). (PDF) [file pntd.0012157.s011.pdf]

Supporting Information for *Influence of hydrometeorological risk factors on child diarrhea and enteropathogens in rural Bangladesh*

S10 Figure. Predicted diarrhea and enteropathogen prevalence by vapor pressure deficit

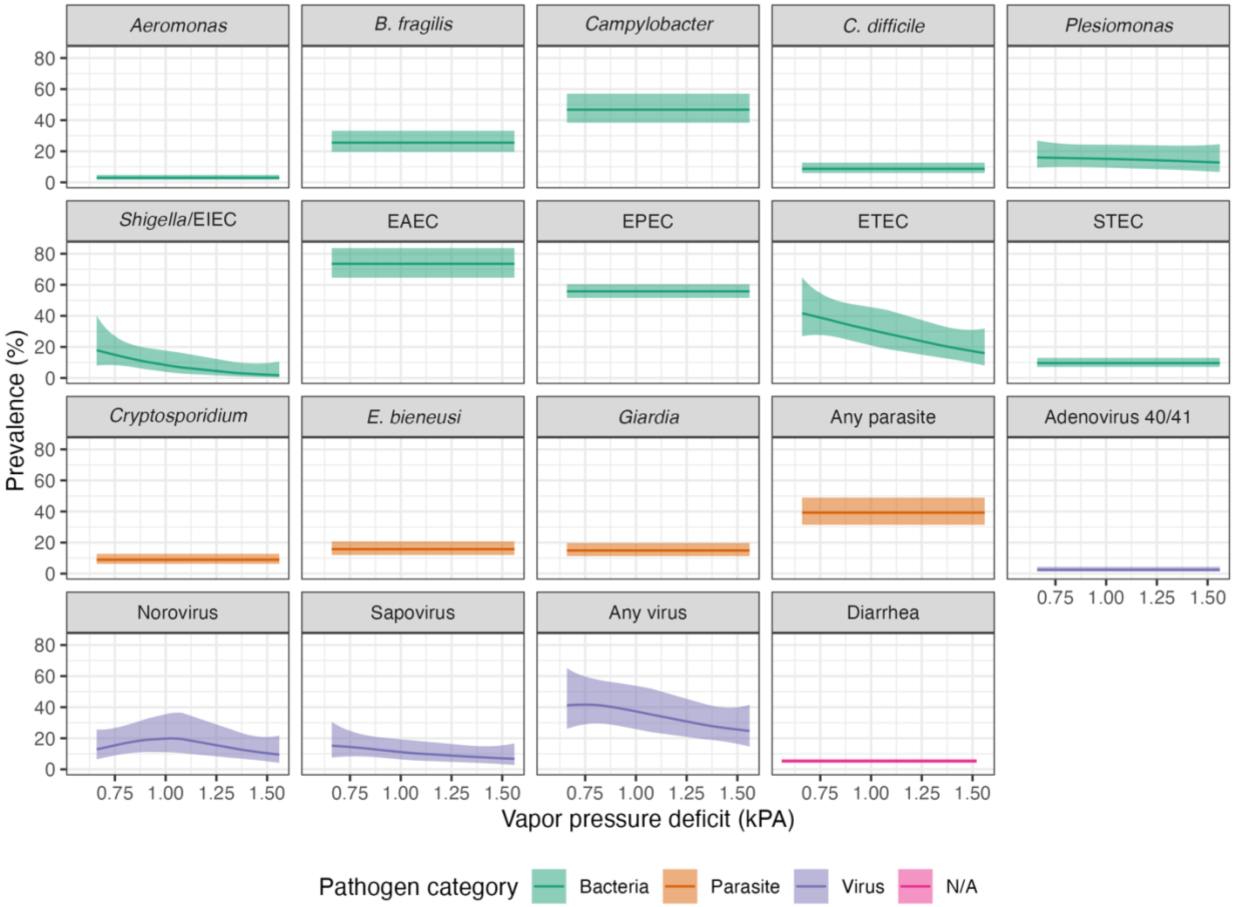

All panels present adjusted models; shaded bands indicate simultaneous 95% confidence intervals accounting for clustering. Pathogen models included measurements in children approximately 14 months of age in the control, combined water + sanitation + handwashing (WASH), nutrition, and combined nutrition + WASH arms of the original trial. Diarrhea model includes measurements in children aged 6 months - 5.5 years in the control arms in the original trial. Prevalence estimates were predicted under conditions which held all adjustment covariates at fixed representative values (see Methods for details).
