# Supplementary material for: Influence of hydrometeorological risk factors on child diarrhea and enteropathogens in rural Bangladesh
Source: PLoS Negl Trop Dis. 2024 May 13;18(5):e0012157. doi: 10.1371/journal.pntd.0012157 (PMC11115220; doi:10.1371/journal.pntd.0012157)
Supplement: S11 Fig — Data are from adjusted models and include measurements in children aged 6 months—5.5 years in the control arms in the original trial. Panels A) and B) Error bars present 95% confidence intervals adjusted for clustering and the x-axis is on the log scale. Panel A) shows an indicator variable for heavy rainfall (total weekly precipitation > 80th (17mm) or 90th (29mm) percentile during the study period or for above median (13mm), 75th (58mm) or 90th (105mm) percentile average weekly precipitation as the independent variable. Panel B) The independent variable was an indicator for whether the proportion of pixels with surface water within 250m, 500m, 750m of each household was above or below the median or a categorical variable for tertiles of distance from each household to the nearest surface water (<165m; 165m to <316m, ≥316m). Panel C) Shaded bands indicate simultaneous 95% confidence intervals accounting for clustering. Prevalence estimates were predicted under conditions which held all adjustment covariates at fixed representative values (see Methods for details). No significant results were observed for other temperature variables or vapor pressure deficit. (PDF) [file pntd.0012157.s012.pdf]

Supporting Information for *Influence of hydrometeorological risk factors on child diarrhea and enteropathogens in rural Bangladesh*

S11 Figure. Interaction between age category and hydrometeorological risk factors on childhood diarrhea prevalence

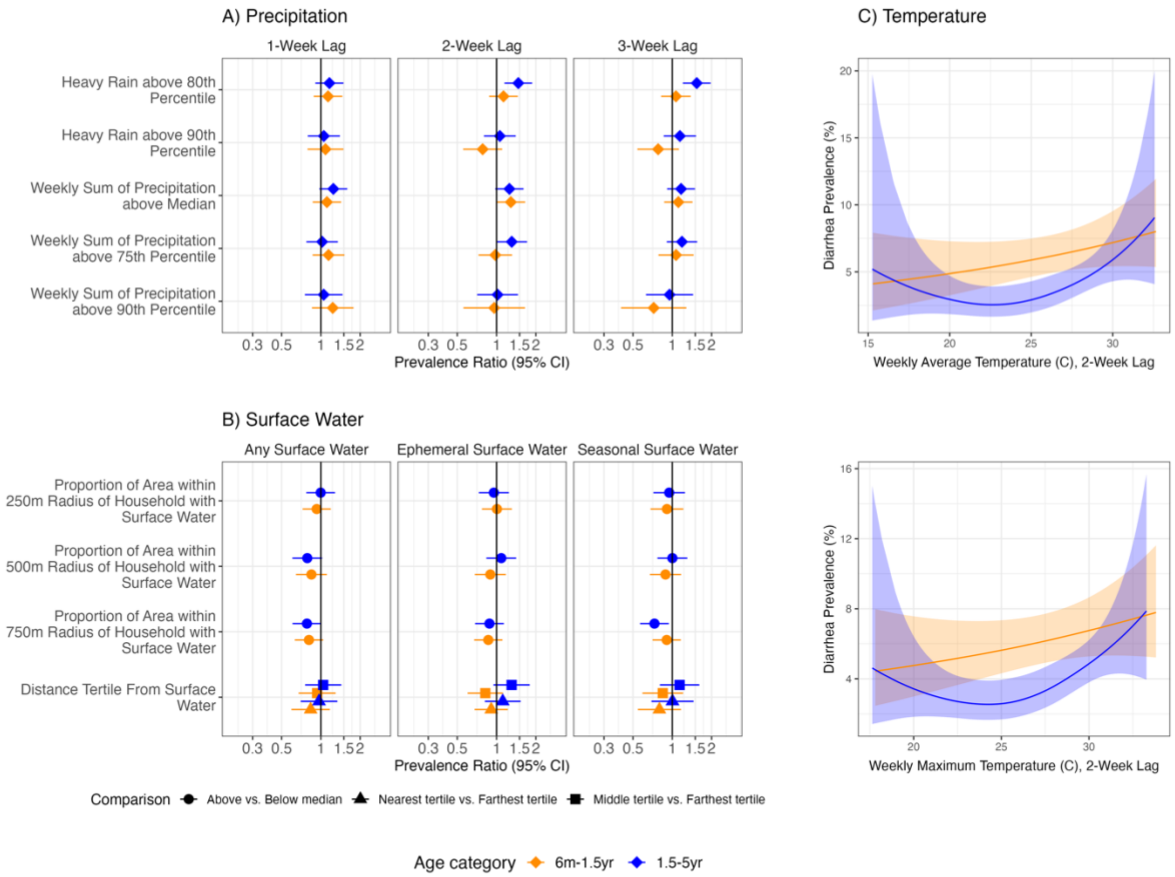

Data are from adjusted models and include measurements in children aged 6 months - 5.5 years in the control arms in the original trial. Panels A) and B) Error bars present 95% confidence intervals adjusted for clustering and the x-axis is on the log scale. Panel A) shows an indicator variable for heavy rainfall (total weekly precipitation > 80th (17mm) or 90th (29mm) percentile during the study period or for above median (13mm), 75th (58mm) or 90th (105mm) percentile average weekly precipitation as the independent variable. Panel B) The independent variable was an indicator for whether the proportion of pixels with surface water within 250m, 500m, 750m of each household was above or below the median or a categorical variable for tertiles of distance from each household to the nearest surface water (<165m; 165m to <316m, ≥316m). Panel C) Shaded bands indicate simultaneous 95% confidence intervals accounting for clustering. Prevalence estimates were predicted under conditions which held all adjustment covariates at fixed representative values (see Methods for details). No significant results were observed for other temperature variables or vapor pressure deficit.
