# Supplementary material for: Influence of hydrometeorological risk factors on child diarrhea and enteropathogens in rural Bangladesh
Source: PLoS Negl Trop Dis. 2024 May 13;18(5):e0012157. doi: 10.1371/journal.pntd.0012157 (PMC11115220; doi:10.1371/journal.pntd.0012157)
Supplement: S12 Fig — All panels present adjusted models for weekly minimum, mean, or maximum temperature with the indicated lag period; shaded bands indicate simultaneous 95% confidence intervals accounting for clustering. Includes measurements in children aged 6 months—5.5 years in the control arms in the original trial. Prevalence estimates were predicted under conditions which held all adjustment covariates at fixed representative values (see Methods for details). (PDF) [file pntd.0012157.s013.pdf]

S12 Figure. Negative control analysis – bruising and temperature

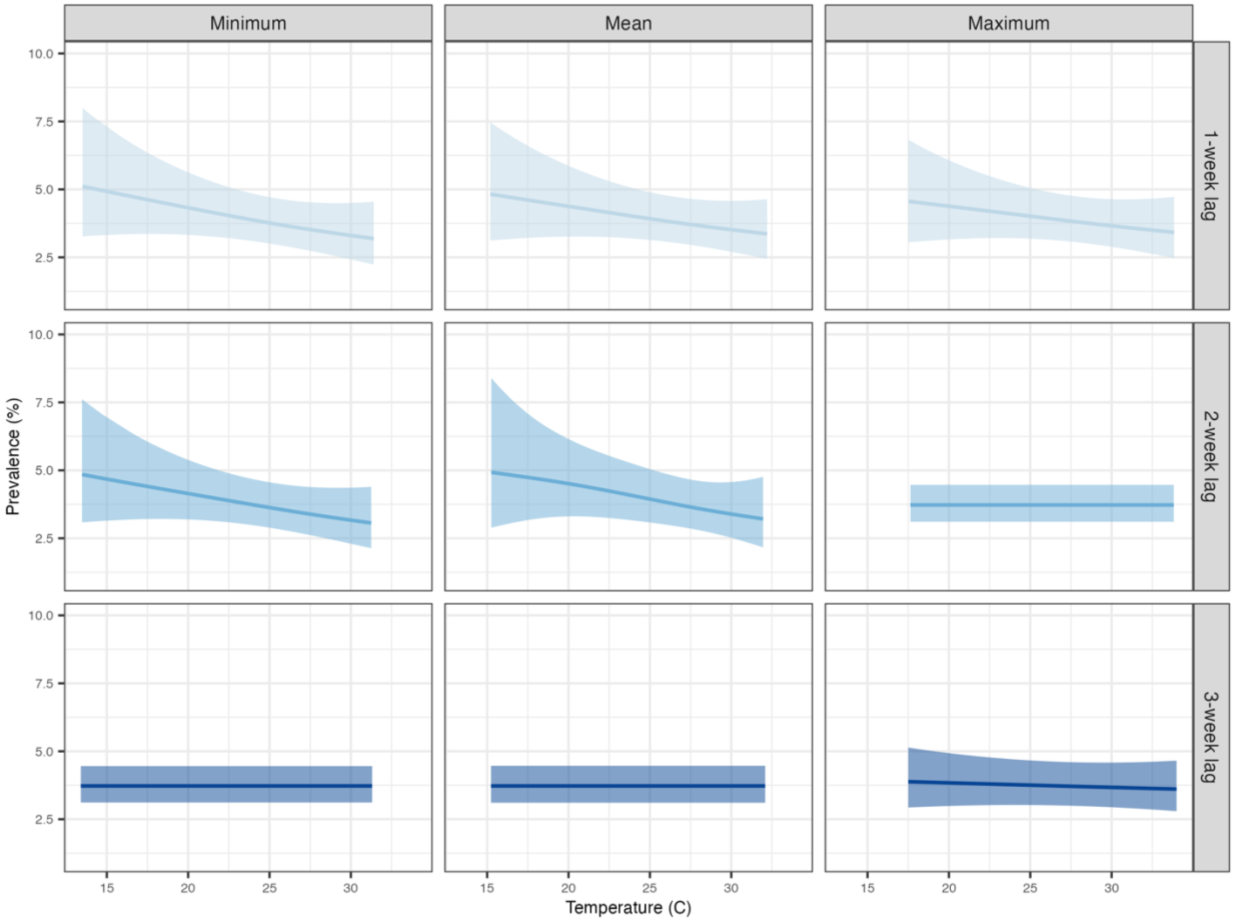

All panels present adjusted models for weekly minimum, mean, or maximum temperature with the indicated lag period; shaded bands indicate simultaneous 95% confidence intervals accounting for clustering. Includes measurements in children aged 6 months - 5.5 years in the control arms in the original trial. Prevalence estimates were predicted under conditions which held all adjustment covariates at fixed representative values (see Methods for details).
