# Supplementary material for: Influence of hydrometeorological risk factors on child diarrhea and enteropathogens in rural Bangladesh
Source: PLoS Negl Trop Dis. 2024 May 13;18(5):e0012157. doi: 10.1371/journal.pntd.0012157 (PMC11115220; doi:10.1371/journal.pntd.0012157)
Supplement: S1 Table — (PDF) [file pntd.0012157.s014.pdf]

Supporting Information for *Influence of hydrometeorological risk factors on child diarrhea and enteropathogens in rural Bangladesh*

S1 Table. Bacterial enteropathogen prevalence estimates at weekly average temperatures of 19°C and 30°C.

Enteropathogen outcome	Prevalence at 19°C (95% CI)	Prevalence at 30°C (95% CI)
Aeromonas	1.5 (0.1, 21.2)	5.6 (3.1, 10.3)
B. fragilis	27.5 (22.9, 33.0)	27.5 (22.9, 33.0)
C. difficile	9.5 (5.6, 16.0)	8.5 (5.4, 13.4)
Campylobacter	52.9 (45.1, 62.0)	52.9 (45.1, 62.0)
EAEC	77.5 (67.1, 89.6)	80.5 (72.5, 89.4)
EPEC	55.7 (51.4, 60.3)	55.7 (51.4, 60.3)
ETEC	30.9 (19.7, 48.6)	48.2 (37.9, 61.2)
Plesiomonas	16.0 (12.1, 21.3)	16.0 (12.1, 21.3)
STEC	6.8 (3.5, 13.2)	14.1 (10.9, 18.2)
Shigella/EIEC	8.4 (3.7, 19.0)	16.4 (10.9, 24.7)

Includes measurements in children approximately 14 months of age in the control, combined water + sanitation + handwashing (WASH), nutrition, and combined nutrition + WASH arms of the original trial.

The following covariates were pre-screened with a likelihood ratio test and those associated with the outcome (p-value < 0.1) were included in adjusted models: child age, sex, antibiotic use in the previous 7 days, household wealth quartile, whether the household received a WASH intervention, and whether the household received a nutrition intervention.

The prevalence estimates were predicted using adjusted models with a 1-week lag under conditions which held all adjustment covariates at fixed representative values. Continuous covariates were fixed at the median across all samples, WASH and nutrition interventions were fixed at receiving the intervention, household wealth was fixed at the lowest quartile, antibiotic use was fixed at no antibiotic use reported in the previous 7 days, and sex was fixed at male.
