# Supplementary material for: Influence of hydrometeorological risk factors on child diarrhea and enteropathogens in rural Bangladesh
Source: PLoS Negl Trop Dis. 2024 May 13;18(5):e0012157. doi: 10.1371/journal.pntd.0012157 (PMC11115220; doi:10.1371/journal.pntd.0012157)
Supplement: S3 Table — (PDF) [file pntd.0012157.s016.pdf]

Supporting Information for *Influence of hydrometeorological risk factors on child diarrhea and enteropathogens in rural Bangladesh*

S3 Table. Adjusted prevalence ratios for caregiver-reported child bruising in the prior 7 days

Risk factor	N	Prevalence exposed	Prevalence unexposed	Unadjusted prevalence ratio (95% CI)
Heavy Rain				
1-Week Lag	6579	2.58%	3.41%	0.94 (0.71, 1.25)
2-Week Lag	6579	3.37%	3.21%	0.84 (0.63, 1.12)
3-Week Lag	6579	2.33%	3.43%	0.85 (0.64, 1.14)
Above Median Weekly Sum of Precipitation				
1-Week Lag	6579	2.97%	3.54%	0.85 (0.65, 1.12)
2-Week Lag	6579	2.92%	3.57%	0.82 (0.62, 1.07)
3-Week Lag	6579	2.94%	3.56%	0.79 (0.60, 1.04)
Distance Tertile from Any Surface Water				
Close vs. Far	6579	3.61%	3.54%	0.88 (0.64, 1.21)
Medium vs. Far	6579	2.54%	3.54%	0.66 (0.47, 0.94)
Distance Tertile from Seasonal Surface Water				
Close vs. Far	6579	3.29%	3.15%	0.88 (0.63, 1.24)
Medium vs. Far	6579	3.28%	3.15%	1.01 (0.73, 1.41)
Distance Tertile from Ephemeral Surface Water				
Close vs. Far	6579	3.14%	3.20%	0.87 (0.62, 1.22)
Medium vs. Far	6579	3.39%	3.20%	1.05 (0.76, 1.46)
Above Median Proportion of Surface Water Near Household				
Any Surface Water, 250m radius	6579	3.22%	3.26%	0.88 (0.67, 1.16)
Seasonal Surface Water, 250m radius	6579	3.40%	3.14%	0.94 (0.71, 1.25)
Ephemeral Surface Water, 250m radius	6579	3.23%	3.24%	0.88 (0.65, 1.18)
Any Surface Water, 500m radius	6579	3.03%	3.46%	0.82 (0.62, 1.07)
Seasonal Surface Water, 500m radius	6579	3.18%	3.30%	0.90 (0.68, 1.18)
Ephemeral Surface Water, 500m radius	6579	3.13%	3.35%	0.88 (0.67, 1.16)
Any Surface Water, 750m radius	6579	2.95%	3.54%	0.77 (0.59, 1.01)
Seasonal Surface Water, 750m radius	6579	3.16%	3.32%	0.85 (0.65, 1.12)
Ephemeral Surface Water, 750m radius	6579	3.16%	3.32%	0.84 (0.64, 1.10)

Includes measurements in children aged 6 months - 5.5 years in the control arms in the original trial
