# Supplementary material for: Influence of hydrometeorological risk factors on child diarrhea and enteropathogens in rural Bangladesh
Source: PLoS Negl Trop Dis. 2024 May 13;18(5):e0012157. doi: 10.1371/journal.pntd.0012157 (PMC11115220; doi:10.1371/journal.pntd.0012157)
Supplement: Attachment — Submitted filename: Revision PNTD-D-23-01115.docx [file pntd.0012157.s017.docx]

Influence of weather and environmental risk factors on child diarrhea and enteropathogens in rural Bangladesh

I believe that the theme of the work is relevant because it deals with prevalent pathologies of importance in public health and that affect a vulnerable population. However, below I detail some comments and suggestions that I believe will improve the interpretation of the study.

**Abstract**

It would be good to describe the study design used in this work.

**Introduction**

The introduction is well presented, addressing the issue of diarrhea and the influence of climatic and environmental factors.

The general objective is well stated. However, it would be appropriate to incorporate some prediction of what they would expect to find considering the prevalence of diarrhea and enteroparasites and the climatic and environmental characteristics of the country.

**Methods**

The methodology lacks clarity and structure.

First I suggest describing at the beginning of the methodology the study design used in order to correctly interpret the association measures evaluated in the study.

I suggest adding information on the country, what percentage of the population is represented in the sample of this research, as well as the representativeness of each of the provinces sampled.

I suggest incorporating more information about the climate of the country and the provinces of the study. For example, the monsoon from June to September that often causing flooding, Hurricane season can occur from May to June and October to November, month with high temperatures and humidity and the period of time with major storms.

Why were trials conducted on pregnant women?

I consider that Figure 1 should show the provinces sampled with the continental location of Bangladesh and the left panel I suggest to put it in a separate figure later when the associations with climate are presented.

Why was the subsample taken in that year (2014)?

Although the sampling information is detailed in supplementary material, I suggest clarifying some issues in the body of the manuscript:

1º At what scale was the climate data worked on?

2ºHow many weather stations do you work with?

3º At what time of the year were the samples taken, given the intra-annual climatic variability?

4º In the case of the environmental variable: only natural or artificial water bodies were recorded (for example, ponds in parks, drains or containment pools).

Proximity to sources of garbage accumulation could have been another interesting environmental variable to take into account and open dumps can be recorded through spectral signatures as well.

Line 212: “We assessed effect modification for the diarrhea outcome by child age”. Why?

**Results**

The results of the participant and sample characteristics detailed in Table 1 are clear. However, the methodology regarding characteristics and sampling (e.g. time of year) is not equally clear in the text (without the need to refer to the Appendix).

Line 232. “The study period spanned three rainy seasons (empirically determined as the period when the 5-day rolling average rainfall was ≥10mm/day) from 2013-2015”: this information should be moved to Methodology.

**Discussion**

Avoid very long sentences:

Line 344. Finish a sentence and start a new one. “ …in rural Bangladesh, we found… “ **replaced by** “…in rural Bangladesh. We found…”

Line 346. Finish a sentence and start a new one. “ … pathogens, and surface water

… “ **replaced by** “…pathogens. Surface water…”.

Line 347. “Our findings suggest that 348 under projected increases in temperature (mean increase of 1° C) and precipitation (mean 349 increase of 6%) from 2021-2050 in Bangladesh due to climate change (33), there would be small 350 increases in the prevalence of diarrhea and certain enteric pathogen infections.” This sentence is very long and I do not think it is relevant in this paragraph where the main findings are summarized.
