# Supplementary material for: Influence of hydrometeorological risk factors on child diarrhea and enteropathogens in rural Bangladesh
Source: PLoS Negl Trop Dis. 2024 May 13;18(5):e0012157. doi: 10.1371/journal.pntd.0012157 (PMC11115220; doi:10.1371/journal.pntd.0012157)
Supplement: Attachment — Submitted filename: PNTD-D-23-01115_Grembi_ReviewerResponse.docx [file pntd.0012157.s018.docx]

We thank the editors and reviewers for their thoughtful comments and have incorporated their feedback in a revised version of our manuscript. Below, we provide point-by-point responses to each comment and list edits that were made in response to the feedback. The listed line numbers correspond to the manuscript version with tracked changes.

**Methods**

Reviewer #1:

1. The study objectives are clear and the methods are appropriate and well-presented.

Response: We are pleased the reviewer found our methods appropriate and clearly presented.

2. Line 202-203:  I am not sure I agree with your conclusion that pair-wise correlations between weather exposures were not strong and thus not necessary to adjust individual exposure models for other weather exposures. This makes sense for surface water distance and perhaps also for vapor pressure deficit, but I don’t think it makes sense for temperature and precipitation. I would consider correlation coefficients around 0.6-0.7 (as you show in Fig S1) to be moderate or strong, and this makes sense - temperature and precipitation are typically correlated. I would consider looking at the effects of temperature and precipitation in combined models, or address this further and provide a justification for not doing so.

Response: We thank the reviewer for noting the strong correlation between temperature and precipitation. In the updated manuscript, we include temperature and precipitation in a combined model, as well as models that include an interaction term. The text has been updated to reflect the new analyses.

Lines 395-446: *“Because pair-wise correlations between temperature and precipitation* ***were strong*** *(S1 Fig),* ***we evaluated temperature exposure models with and without inclusion of precipitation as an additional covariate. We also assessed effect modification of temperature by precipitation using a model with both exposures and an interaction term. Neither vapor pressure deficit nor surface water displayed correlations with other hydrometeorological variables, so we did not adjust these exposure models for other exposures.****”*

3. If you do include temp and precip in combined models, you might also consider testing whether the inclusion of an interaction term between temp and precip improves the model.

Response: We evaluated models that contained both temperature and precipitation exposures and found very few instances where the inclusion of an interaction term improved the model fit (as measured by AIC). Data are presented in a new S2 Table and the following text has been added to the results section:

Lines 778-782: *“****Due to the strong correlation between temperature and precipitation, we included models with both temperature and precipitation variables. Models which included both variables often had improved model fit (lower Akaike Information Criterion) over models with only temperature. There were few instances in which models were improved by the inclusion of an interaction term (S2 Table).”***

4. How did you select these exposure variables? E.g. duration of lag periods, heavy rainfall as >80th percentile, weekly precip over the median. Recommend including a justification. Are the results consistent if these specifications change? It might be valuable to include sensitivity analyses in the supplemental. I see you analyzed 1, 2, and 3 week lags. What about the choice of the 80th percentile and the median precipitation?

Response: We initially chose an 80th percentile (17mm) threshold for heavy rainfall and a median 7-day sum of precipitation (13mm) to minimize data sparsity. Per the reviewer’s suggestion, we have added a sensitivity analysis to the supplemental materials. For heavy rainfall we now also include prevalence ratio estimates for heavy rain vs no heavy rain using a higher 90^th^ percentile (29mm) threshold. For total 7-day precipitation, we now also include prevalence ratio estimates for high weekly precipitation vs not high weekly precipitation using 75^th^ (58mm) and 90^th^ (105mm) percentile thresholds. Using the 90^th^ percentile threshold for heavy rain, we observed that the point estimates were similar but attenuated for most outcomes except diarrhea where the association was completely lost. For total 7-day precipitation using the 75^th^ and 90^th^ percentile cutoffs results were similar for viruses, and similar but attenuated for bacteria and parasites, except for *E. bieneusi* which had a lower prevalence using both the 75^th^ and 90^th^ percentile cutoffs with a 1-week lag. Again, the association was lost for the diarrhea outcome. We have included these sensitivity analyses in the supplemental materials for heavy rainfall (S7 Fig) and for weekly precipitation (S6 Fig). Additionally, we have added an explanation and citations for the choice of lag periods.

Lines 293-300 (Methods): *“****As diarrhea was measured at a weekly resolution (any diarrhea in the previous 7 days), we included 1- to 3-week lags for diarrhea and 0- to 3-week lags for pathogen outcomes per previous studies showing these as relevant lag times between temperature or precipitation and either diarrhea or enteric pathogens (9,18,37,38). The lag periods include expected time for microbial growth/death due to weather (e.g. temperature and humidity), mobilization and transport of enteropathogens persisting in the environment (e.g. after precipitation), and post-exposure enteropathogen-specific incubation times within the host to reach detectable levels and/or initiate a diarrheal response (S3 Appendix).****”*

Lines 317-330 (Methods): *“****Precipitation thresholds were based on the percentile of all daily totals (across all study years and not season-specific) in order to maximize the generalizability of our results. Each variable was calculated with 1-3-week lags for diarrhea and 0-3-week lags for pathogen outcomes. As a sensitivity analysis for the thresholds to define the categorical variables, we also include a heavy rainfall variable calculated using the 90^th^ percentile (29mm), and weekly sum of precipitation above the 75^th^ (58mm) and 90^th^ (105mm) percentiles****.”*

Lines 599-602 (Results): *“Above-median weekly total precipitation (>13mm) was associated with higher diarrhea prevalence (PR = 1.21; 95% CI 0.98, 1.51; adjusted PR [aPR] = 1.29, 95% CI 1.07, 1.55) using a 2-week lag, but not using 1- or 3-week lags (Fig 4A)* ***nor when using higher threshold cutoffs (75^th^ and 90^th^ percentile, S6 Fig).”***

Lines 606-619 (Results): *“For bacteria, above-median weekly precipitation was associated with higher prevalence of enterotoxigenic E. coli (ETEC), STEC, Shigella/EIEC, Campylobacter, and Aeromonas (Fig 4D).* ***With higher percentile cutoffs, results were similar for viruses, and similar but attenuated for bacteria and parasites except for E. bieneusi, for which increased precipitation was associated with a lower prevalence using both the 75^th^ and 90^th^ percentile cutoffs with a 1-week lag (S6 Fig).*** *Heavy rainfall (>80th percentile, 17mm) on a single day was associated with higher prevalence of diarrhea (aPR = 1.31, 95% CI 1.09, 1.57; 2-week lag), Cryptosporidium, adenovirus 40/41, Aeromonas, Campylobacter, Shigella/EIEC, ETEC, and STEC and lower prevalence of norovirus and sapovirus (S7 Fig).* ***Results were similar with a 90^th^ percentile cutoff threshold for all outcomes except diarrhea where the association was lost (>29mm; S7 Fig).*** *Results varied by lag period, and the lag period expected to be most important based on enteropathogen-specific incubation times (S3 Appendix) were often not the most strongly associated with above-median precipitation or heavy rainfall (Figs 4B-D, S6 Fig,* ***S7 Fig****).”*

5. Line 323: How were 250m, 500m, 750m distance thresholds selected?

Response: These thresholds were determined by a combination of the resolution of the data (30m) and distances that we felt captured risks associated with the potential for flooding in the household area during heavy rains. There is no precedent for this in the literature that we could find. We have added the following clarification to the manuscript:

Line 344-345: “***The distance thresholds were determined to capture risks associated with the potential for flooding in the household environment during heavy rainfall.****”*

6. Line 232: Could you provide a citation or justification for this method of determining the rainy season?

Response: Most prior studies use fixed periods sometime between June-October to define the rainy season in Bangladesh, but we developed a data-driven definition of seasonality to account for variations in timing for each study year. In Bangladesh, precipitation is heavily concentrated during the rainy season and the first instance of continuous rainfall typically marks the start of the season; using rolling averages of rainfall allowed us to identify when this period started. Based on observed precipitation values in the study region, we selected a threshold of 10 mm that captured the beginning of the period of consistent rainfall during the monsoon months, as shown below and in Figure A1 of our pre-analysis plan on OSF (<https://osf.io/f9cza>), where green marks the start of the season and red marks the end of the season. We have added details in the methods section to justify this choice.

Line 330-335: “***Although prior studies have used different fixed periods between the months of June and October as the rainy season in Bangladesh (30,41,42), we observed variations in the start and end of the rainy period in different years of our study, with substantial rainfall before June in every year. Therefore,*** *we determined the rainy season empirically* ***(10),*** *defined as the continuous period during which the 5-day rolling average of daily precipitation was ≥10mm/day.”*

30.	Ali M, Emch M, Donnay JP, Yunus M, Sack RB. Identifying environmental risk factors for endemic cholera: a raster GIS approach. Health & Place. 2002 Sep;8(3):201–10.

41.	Chowdhury FR, Ibrahim QSU, Bari MS, Alam MMJ, Dunachie SJ, Rodriguez-Morales AJ, et al. The association between temperature, rainfall and humidity with common climate-sensitive infectious diseases in Bangladesh. PLOS ONE. 2018 Jun 21;13(6):e0199579.

42.	Hashizume M, Armstrong B, Wagatsuma Y, Faruque ASG, Hayashi T, Sack DA. Rotavirus infections and climate variability in Dhaka, Bangladesh: a time-series analysis. Epidemiology & Infection. 2008 Sep;136(9):1281–9.

10.	Chao DL, Roose A, Roh M, Kotloff KL, Proctor JL. The seasonality of diarrheal pathogens: A retrospective study of seven sites over three years. PLOS Neglected Tropical Diseases. 2019 Aug 15;13(8):e0007211.

7. The authors have clearly made an effort to make their research process transparent (publicly available deidentified data, code, and pre-specified analysis plan). This is commendable. For the code - you state that code is available at <https://github.com/jgrembi/WBB-weather-diarrhea-pathogens>, but I get an error message when I try to access this. I am assuming you intend to make it available post publication?

Response: We have updated the link with a publicly available version: https://github.com/jgrembi/wbb-weather-diarrhea-enteropathogens. We apologize for the inconvenience.

Reviewer #2:

8. The manuscript reports an analysis of secondary data that was originally collected to test the efficacy of WASH and nutrition interventions on childhood diarrhea and growth. The objective is stated clearly at the end of the introduction, though it is not restated as a testable hypothesis, perhaps because of the complexity of the analysis and the multiple pairwise exposure/outcome associations tested. If stating a hypothesis is a requirement, perhaps it could be something like “there are pathogen-specific differences in the direction, magnitude, and significance of associations between environmental exposures and enteropathy outcomes”.

Response: We have included the following hypothesis at the end of the introduction:

Line 189-190: “***We hypothesize that there are pathogen-specific differences in the direction, magnitude, and significance of associations with hydrometeorological exposures.****”*

9. Broadly speaking, the study design is appropriate and follows approaches that have precedents in prior literature. The authors match publicly available hydrometeorological exposure data spatiotemporally with epidemiological outcome data collected by the trial. Unadjusted and adjusted associations are characterized by fitting GAM models, while giving appropriate consideration to lagged effects, spatial autocorrelation, and potential confounding. Notably, they link to their pre-analysis plan and document deviations from it in an appendix, a level of transparency that is rare.

Response: We appreciate the referee’s enthusiasm for our commitment to transparent and rigorous research methodologies and have endeavored to address their comments below.

10. I think that the structure of the dataset and its implication for the model specification could be better explained. It seems to me that the data is hierarchical, with stool samples/diarrhea episodes from multiple rounds nested within subjects (children), subjects nested within compounds, and compounds within villages (clusters). Random intercepts were used in the models to account for this clustering but at different levels depending on the outcome (compound-level for diarrhea, village-level for enteropathogens). I’m sure there were good reasons for treating the outcomes differently, but the justification is not clear as currently written. Were subject-level random effects specified in all models?

Response: The referee is correct that we accounted for the hierarchical clustering of the data differently in the diarrhea and pathogen analyses. We have clarified this in the text and provided justification for treating the outcomes differently. Subject-level random effects were not used. The updated text reads:

Lines 371-377: “***Due to multiple measurements within a study compound in the diarrhea dataset (e.g. index children and siblings sampled within a single compound on the same day and data collected over multiple years),*** *we included random intercepts for study compound for diarrhea models.* ***In the pathogen dataset, only a single measurement was collected for each index child thus we included*** *random intercepts for study cluster for pathogen outcomes to account for village-level clustering.”*

11. Were the stool samples in which enteropathogens were diagnosed all collected during diarrheal episodes, or were there samples from asymptomatic children?

Response: Stool samples were not specifically targeted for collection during diarrheal episodes, they were collected from children at the time of an annual household visit, during which approximately 14% (n=214) reported diarrheal symptoms in the previous 7 days. Thus, a majority of samples (86%) were collected from asymptomatic children. However, there are strong associations between asymptomatic enteropathogen carriage and linear growth faltering, and given that the majority of our study population falls under this latter category and suffers from impaired growth, we feel it is relevant to evaluate pathogen carriage, regardless of the presence of diarrheal symptoms. We have clarified this in the text as follows:

Lines 255-259: “***Stool samples were collected as part of a routine household visit regardless of children’s symptom status (14% of children reported diarrheal symptoms in the previous 7 days at the time of stool collection). We chose to collect stool from both asymptomatic and symptomatic children because prior literature has shown the importance of asymptomatic pathogen carriage on growth faltering (2,32,33).***”

12. The use of child bruising as a negative control outcome is appropriate since it can’t plausibly be linked with the exposures or outcomes, however I disagree that the reason for this is “to detect potential misclassification of diarrhea”. The purpose of a negative control outcome is to infer causality with greater confidence (by demonstrating the specificity of the association) and to rule out measured and unmeasured confounding as an explanation for any statistically significant main associations observed (i.e., if an association between an exposure and an NCO is significant, then the main association may be spurious).

Response: We agree with the reviewer that a negative control outcome (NCO) is useful to demonstrate specificity of the association and attempt to infer causality with greater confidence. NCOs can also detect measurement (information) biases (e.g. misclassification), so we have revised the sentence accordingly:

Lines 267-269: *“To detect potential* ***unmeasured confounding or outcome measurement bias in the*** *diarrhea* ***analysis, we repeated the analysis using c****aregiver-reported child bruising in the past 7 days as a negative control outcome (35,36).”*

13. I think that the authors’ choice of climate data sources requires some justification. FLDAS was generated for Central Asia, specifically Afghanistan, and not for Bangladesh. The resolution of the NOAA precipitation dataset used is extremely coarse at 55km2. It’s difficult to tell from figure 1 (perhaps include a scalebar) but probably all the clusters are covered by a handful of grid squares, while precipitation varies on a very small scale. The CHIRPS precipitation dataset (<https://doi.org/10.1038/sdata.2015.66>) has much higher resolution, but also consider the ERA5 dataset (<https://doi.org/10.1002/qj.3803>), which has been used in epidemiological studies of infectious syndromes (<https://doi.org/10.1038/s41467-021-25914-8>) and includes many of the variables relevant to this analysis (<https://doi.org/10.1038/s41597-023-02276-y>), with the advantage that they are from a single source and mutually consistent.

Response: We appreciate the reviewer’s suggestion for alternative hydrometeorological data sources. The FLDAS dataset covers a "Central Asia" region that is defined by the bounds 30 to 100°E, 21 to 56°N, which contains our study area. It is our understanding that the model was developed and validated for that entire region, thus we believe our analysis using this data is valid. We erroneously reported the use of the NOAA precipitation dataset as specified in our pre-analysis plan. However, due to the poor spatial resolution noted by the reviewer, prior to manuscript submission we switched to the GloH2O dataset for precipitation which includes daily measurements at 0.1 degree (~10km) spatial resolution, which has better spatial resolution than the ERA5 dataset (31km resolution). We have updated the methods section and Supplemental materials (S1 Appendix. Deviations from pre-analysis plan) to reflect the precipitation dataset that was actually used for the analyses. We have also included a scalebar in Figure 1, per the reviewer’s recommendation.

Lines 311-313: “*We obtained* ***0.1 degree (~10 km) resolution*** ***daily precipitation data from the Multi-Source Weighted-Ensemble Precipitation dataset from GloH2O which merges gauge, satellite, and reanalysis data and corrects for bias (40).****”*

*S1 Appendix:*

1) *“****We pre-specified the use of the National Oceanic and Atmospheric Administration/OAR/ESRL Physical Science Laboratory dataset (Xie 2007, Chen 2008) for precipitation data. However, given the poor spatial resolution (~55km), we instead used the GloH2O Multiple-Source Weighted-Ensemble Precipitation dataset (Beck 2019). This dataset has ~10km spatial resolution on daily precipitation data that incorporates gauge, satellite, and reanalysis data, and corrects for bias.****”*
14. The explanation for the aggregation and lagging of the meteorological exposures is a little unclear. Was the resolution of both the enteropathogen and diarrhea variables daily? At points it sounds like for diarrhea it was weekly. In any case, I think you should state in the main text that two of the main exposures had daily resolution (temperature and precipitation) and the other had monthly resolution (surface water and VPD). Those with daily resolution were aggregated in different ways over a 7-day window (as well as a 30-day window for temperature), and lagged by 7, 14, and 21 days. So, if t0 is the day of outcome ascertainment, then one of the exposures was the average temperature from t-7 to t-14 (see notation used in 10.1029/2021GH000452). The monthly exposures were linked to the outcomes by calendar month and year of outcome ascertainment (without lagging). If I’ve understood this correctly, then I think it might be possible to tighten this explanation. This is especially important for the surface water data as I expect it will not be familiar to readers.

Response: The resolution of the diarrhea variable was weekly (any diarrhea reported within the previous 7 days) while enteropathogens were measured at a single timepoint. We have added detail as suggested by the reviewer to the section describing the hydrometeorological variables. The new text reads:

Lines 274-296: “***Two of the exposures (temperature and precipitation) had daily resolution data and the other (surface water and humidity) had monthly resolution. Those with daily resolution were aggregated in different ways over a 7-day window (as well as a 30-day window for temperature) and lagged by 0, 1, 2, or 3 weeks (corresponding to the periods 1-7, 8-14, 15-21, and 21-28 days before outcome measurement). As diarrhea was measured at a weekly resolution (any diarrhea in the previous 7 days), we included 1- to 3-week lags for diarrhea and 0- to 3-week lags for pathogen outcomes per previous studies reporting these as relevant lag times between temperature or precipitation and either diarrhea or enteric pathogens (9,18,37,38).”***

15. Some additional details of the modeling approach appear to be missing. Please state the link function and family of the model used. From what I can infer, the outcomes were binary (presence or absence of a diarrhea episode/enteropathogen), so would that have been a log link and binomial family? Were prevalence and prevalence ratios approximated from transformations of odds ratios or risk ratios?

Response: We agree the information on the family and link function should be included. We pre-specified a binomial family with a logit link for binary outcomes. However, to estimate the prevalence ratio, we instead used modified Poisson regression (log link) because many of our outcomes were not rare (e.g. EAEC was detected in 76% of children). We have added this to the list of deviations from the pre-analysis plan in Appendix S1 and the following text has been added to the manuscript along with a reference for the use of Poisson regression to estimate prevalence ratios:

S1 Appendix.

6) *“****We pre-specified the use of generalized additive mixed models with a binomial family and logit link for binary outcomes. However, odds ratios obtained from binomial logit models are not a good estimate of the prevalence ratio when the outcome is common, which is the case for many of our pathogen outcomes (e.g. EAEC was detected in 76% of children). Thus, we instead used Poisson regression (log link) for all outcomes, which provides appropriate estimates of the prevalence ratio for all of our outcomes.”***

Lines 351-369: *“We used generalized additive mixed models* ***with a Poisson family with a log link*** *to estimate the relationship between exposures (continuous exposures modeled with cubic splines, categorical exposures modeled as factors relative to a reference category) and outcomes (45–****47****). To estimate simultaneous confidence intervals, we resampled from the variance-covariance matrix under a multivariate normal distribution (48).”*

47. Zou G. A Modified Poisson Regression Approach to Prospective Studies with Binary Data. American Journal of Epidemiology. 2004 Apr 1;159(7):702–6.

16. If I’ve understood appendix 5 correctly, there were a base set of non-environmental exposures which were included in all models to adjust for their potential confounding effects. This is appropriate. Then for each environmental exposure, a single specific other confounder was considered (but not in the case of precipitation and temperature). Since monthly precipitation was considered as a confounder for two exposures, why not fit a fully adjusted model, testing the associations of all 5 risk factors in table S5 in the presence of each other? (The answer to this question may be that you would need a way of choosing which lag and aggregation window length to use for each exposure/outcome pair and this would overcomplicate the analysis).

Response: The reviewer has understood S5 Appendix and correctly assessed that the complexity of lag periods and aggregation windows makes it difficult to test the association of all risk factors in the presence of each other. In the updated manuscript, we have included models with both temperature and precipitation, and evaluated the same models with inclusion of an interaction term. We evaluated all lag periods for weekly and monthly average temperature against each of the precipitation variables with the same lag period. We observed that including both a temperature and precipitation risk factors resulted in slightly improved models (lower AIC) but we rarely identified interaction effects (S2 Table). Because the primary focus of this analysis was to identify specific hydrometeorological risk factors associated with our outcomes, we do not go into detail on the results of these models. The updated text reads:

Lines 778-782: *“****Due to the strong correlation between temperature and precipitation, we included models with both temperature and precipitation variables. Models which included both variables often had improved model fit (lower Akaike Information Criterion) over models with only temperature. There were few instances in which models were improved by the inclusion of an interaction term (S2 Table).****”*

17. Has the distribution of animal ownership by compound been reported anywhere? The use of this variable seems to me to be a bit of a buried lead, and I think a lot of readers would be interested to see the main effect of that variable on the outcomes, or its interaction with the environmental exposures. I do understand, however, that this is beyond the scope of this analysis.

Response: We agree this is a very interesting question. There are different types of animals present in the household environment (cows, goats, chickens, other poultry) which would require specification of both the number and types of animals present. In addition, the number of animals present in the household environment varies over time as families buy and sell animals. Thus, this is a complicated analysis that falls outside the scope of the current manuscript. Others in our group have investigated this and shown that animal feces contribute to fecal contamination of the environment and are an important risk factor for childhood diarrhea (references below).

Ercumen et. al. Animal Feces Contribute to Domestic Fecal Contamination: Evidence from E. coli Measured in Water, Hands, Food, Flies, and Soil in Bangladesh. Environ. Sci. Technol. 2017, 51, 15, 8725-8734

Ercumen et. al. Do Sanitation Improvements Reduce Fecal Contamination of Water, Hands, Food, Soil, and Flies? Evidence from a Cluster-Randomized Controlled Trial in Rural Bangladesh. Environ. Sci. Technol. 2018, 52, 21, 12089-12097.

Kwong et. al. Ingestion of Fecal Bacteria along Multiple Pathways by Young Children in Rural Bangladesh Participating in a Cluster-Randomized Trial of Water, Sanitation, and Hygiene Interventions (WASH Benefits). Environ. Sci. Technol. 2020, 54, 21, 13828-13838.

18. Lines 194 – 195: I’m a little unsure about the justification for dealing with study arm differently for diarrhea and pathogens. Are the results different if you include study arm as a covariate for the diarrhea models, or exclude the intervention arms for pathogens? It seems like a common approach to both outcomes would be fine and would make results more comparable.

Response: We appreciate this comment and agree our justification for this difference should be made clearer in the manuscript. In a parallel analysis, we observed a significant influence of interventions on diarrhea in a seasonally-dependent manner, thus we restricted our analysis of the diarrhea outcome to observations collected at the study baseline (before intervention delivery) or the control arms of the study (for later time points), so there was no study arm to adjust for. We decided to continue with our approach to pool across intervention arms for the pathogen analysis to maximize statistical power as we had a substantially smaller sample size (N=1408 compared to N=7320). We have moved the text describing this justification up to the Outcomes portion of the Methods section alongside where we describe the diarrhea and measurements (it was previously in the Statistical analysis section at the end of the Methods), so it is clear to the reader much earlier that we only included control arm children. We also emphasized the reason for the difference in the Statistical analysis section. The updated text reads:

Lines 224-229 (Outcomes): *“Field workers measured caregiver-reported diarrhea in the previous 7 days (three or more loose or watery stools in a 24-hour period or a single stool with blood) in children 6 months - 5.5 years old (N=4,478 children).* ***Diarrhea analyses were restricted to the control arms of the original trial or data collected before intervention delivery (i.e. intervention arms at the baseline survey) (N=7,320 measurements) because intervention effects on diarrhea are modified by weather (34).****”*

Lines 380-386 (Statistical analysis): *“We adjusted for covariates that were associated with each outcome in our dataset using a likelihood ratio test (p-value < 0.1) (details in S5 Appendix). For pathogen carriage outcomes,* ***we pooled across all study arms in which specimens were collected (N=1,408 measurements) to maximize statistical power and included receipt of WASH or nutrition interventions as covariates; this was not necessary for the diarrhea outcome as only observations from the control arms were used****.”*

19. Lines 199 – 200: I suggest “(continuous exposures modeled with cubic splines, categorical exposures modeled as factors relative to a reference category)”.

Response: We thank the reviewer for this clarification and have modified the text as suggested.

Reviewer #3:

20. The methodology lacks clarity and structure. First I suggest describing at the beginning of the methodology the study design used in order to correctly interpret the association measures evaluated in the study.

Response: We have endeavored to make the methodology as clear as possible by moving most of the information from S2 Appendix to the main body of the manuscript. The following details were added to the main manuscript text (and S2 Appendix has been removed):

Lines 195-213 (Methods – Study design): *“We analyzed data that was collected in a cluster-randomized trial of water, sanitation, handwashing, and nutrition interventions in rural Gazipur, Mymensingh, Tangail, and Kishoreganj districts of Bangladesh (Fig 1) (31). From 2012-2013, the trial enrolled a total of 720 village clusters spanning a geographic area of 12,500 km2. The trial enrolled pregnant women and measured outcomes* ***longitudinally*** *in their children.* ***On average, approximately 10% of the village was enrolled in each cluster (31) and the sample is generalizable to areas of mainland Bangladesh with similar ecological and socioeconomic features. This analysis was restricted to children enrolled in one of two cohorts of the WASH Benefits Bangladesh study: 1) the diarrhea cohort or 2) the enteropathogen cohort. These data span 3 years and were collected in one of six rounds (three rounds for the diarrhea cohort; and three rounds for the enteropathogen cohort which occurred ~6 months after each diarrhea cohort visit for children enrolled in this cohort). Each data collection round lasted roughly one year. Approximately 54 clusters were sampled per month from the diarrhea cohort, and 14 clusters were sampled per month from the enteropathogen cohort in a spatially dependent manner as the study team moved around the study area over the course of a year. Each round of household visits started in the same place and progressed along the same geographic path, thus compounds which were visited during the rainy season in the first year were more likely to be visited in the rainy season in subsequent years.*** *Our pre-analysis plan is available at https://osf.io/f9cza/, and we note deviations from it in S1 Appendix.”*

Lines 224-247 (Methods – Outcomes): *“Diarrhea. Field workers measured caregiver-reported diarrhea in the previous 7 days (three or more loose or watery stools in a 24-hour period or a single stool with blood) in children 6 months - 5.5 years old (N=4,478 children).* ***The diarrhea cohort was restricted to the control arms of the original trial or data collected before intervention delivery (i.e. intervention arms at the baseline survey) (N=7,320 measurements) because intervention effects on diarrhea are modified by weather (34). Diarrhea prevalence was measured in three main rounds from the diarrhea cohort (N = 6,589 observations; May 2012 - July 2013, September 2013 - September 2014, December 2014 - October 2015). This included index children (live births of women pregnant at the time of enrollment), other children living within the same compound that were younger than 3 years at study enrollment, and children within the same household born after the index children. Data on caregiver-reported diarrhea was collected in three additional rounds from children in the enteropathogen cohort (N = 731 observations from index children in the control arm; December 2012 - January 2014, November 2013 - November 2014, and March 2015 - March 2016).”***

Lines 249-259 (Methods – Outcomes): *“Enteropathogens.* ***The enteropathogen cohort included index children from a subsample of clusters that was evenly balanced across the control, WSH, nutrition, and N+WSH arms (allocation ratio 1:1:1:1). Clusters in each arm were selected based on logistical feasibility for specimen collection and transport to a central laboratory.*** *Field workers collected stool samples from these children* ***between November 2013 and November 2014 when they were approximately*** *14 months* ***old and evaluated samples (N=1,408) for 34 enteric viruses, bacteria and parasites using qPCR (details in S2 Appendix). Stool samples were collected as part of a routine household visit regardless of children’s symptom status (14% of children reported diarrheal symptoms in the previous 7 days at the time of stool collection). We chose to collect stool from both asymptomatic and symptomatic children because prior literature has shown the importance of asymptomatic pathogen carriage on growth faltering (2,32,33).”***

21. I suggest adding information on the country, what percentage of the population is represented in the sample of this research, as well as the representativeness of each of the provinces sampled.

Response: For each village-level cluster, our study enrolled ~10% of the compounds in each village on average. We have added this information to the text.

Lines 199-201: *“****On average, approximately 10% of the village was enrolled in each cluster (31) and the sample is generalizable to areas of mainland Bangladesh with similar ecological and socioeconomic features.****”*

22. I suggest incorporating more information about the climate of the country and the provinces of the study. For example, the monsoon from June to September that often causing flooding,  Hurricane season can occur from May to June and October to November, month with high temperatures and humidity and the period of time with major storms.

Response: We have added language describing the seasonal monsoon from June to September to the introduction. Data on temperature, rainfall, and humidity within the 3-year study period is presented in Figure 2 where peak months are evident, and ranges are provided in the results section for each risk factor. Thus, we felt it would be redundant to add that information again elsewhere.

Lines 177-179: *“In addition, due to its low altitude,* ***seasonal monsoon from June-September with resulting flooding****, and high population density, Bangladesh is among the top ten countries most vulnerable to extreme weather events due to climate change (28).”*

23. Why were trials conducted on pregnant women?

Response: Our study focused on data collected from children under five years of age; in the original trials, pregnant women were enrolled in order to identify a birth cohort in which to measure primary outcomes in the first two years of life. Our study merely leveraged data collected under the parent trial and was not specifically targeted to pregnant women.

24. I consider that Figure 1 should show the provinces sampled with the continental location of Bangladesh and the left panel I suggest to put it in a separate figure later when the associations with climate are presented.

Response: We agree that labeling the provinces sampled would be helpful to readers and have added District labels to Figure 1. As the right panels represent diarrhea prevalence across the study area, we felt it was most appropriate to keep them here alongside the other map as opposed to a separate figure. In response to another reviewer’s comment, we have also added letters to distinguish the panels (A and B), scalebars to represent distance, and text in the caption indicating that the blue star represents Dhaka.

25. Why was the subsample taken in that year (2014)?

Enteropathogen data were originally collected for an evaluation of intervention effectiveness at reducing enteropathogens in children in our trial at approximately 14 months of age (reference below). Children were born in late 2012 and early 2013, so most were of the target age between November 2013 and November 2014, thus why the subsample was taken then. We have added the following text to the manuscript to clarify:

Lines 252-257: *“Field workers collected stool samples from these children* ***between November 2013 and November*** ***2014 when they were*** ***approximately*** *14 months* ***old and evaluated samples (N=1,408) for*** *34 enteric viruses, bacteria and parasites using qPCR (details in S2 Appendix).* ***Stool samples were collected as part of a routine household visit regardless of children’s symptom status (14% of children reported diarrheal symptoms in the previous 7 days at the time of stool collection).****”*

Grembi JA, Lin A, Karim MA, Islam MO, Miah R, Arnold BF, et al. Effect of water, sanitation, handwashing and nutrition interventions on enteropathogens in children 14 months old: a cluster-randomized controlled trial in rural Bangladesh. J Infect Dis. 2020; Available from: <https://academic.oup.com/jid/advance-article/doi/10.1093/infdis/jiaa549/5899213>

26. Although the sampling information is detailed in supplementary material, I suggest clarifying some issues in the body of the manuscript:
    1º At what scale was the climate data worked on?

Response: We have included information on the spatial and temporal resolution for each risk factor.

Lines 305-307: “*Using* ***0.001 degree (~1 km) resolution daily*** *near surface air temperature data from the National Aeronautics and Space Administration Famine Early Warning System Network Land Data Assimilation System (FLDAS) Central Asia dataset (39)….”*

Lines 311-313: *“We obtained* ***0.1 degree (~10 km) resolution daily*** *precipitation data from the Multi-Source Weighted-Ensemble Precipitation dataset from GloH2O which merges gauge, satellite, and reanalysis data and corrects for bias (40).”*

Lines 337-338: *“We obtained* ***monthly 30 m*** *resolution surface water data for the period 1984-2020 from the Global Surface Water Explorer (43).”*

Lines 347-348: *“We obtained mean* ***monthly vapor pressure deficit data with 4 km resolution*** *from Terraclimate (44) and measured associations with a continuous measure in kilopascals (kPA).”*

27. 2ºHow many weather stations do you work with?

Response: Our data was based on satellite imagery, not weather stations, with the exception of the precipitation data, which incorporates gauge, satellite and reanalysis data. In the previously submitted version, we had incorrectly specified the data source used for precipitation, which has been updated in this version to reflect the GloH2O dataset.

28. 3º At what time of the year were the samples taken, given the intra-annual climatic variability?

Response: We believe the reviewer is specifically asking about fecal samples used for enteropathogen measurement, as the diarrhea data is presented in Fig 2. Fecal sample collection occurred between Nov 2013 and Nov 2014, so spanned an entire year. We have added this clarification to the text.

Lines 252-255: *“Field workers collected stool samples from these children* ***between November 2013 and November 2014 when they were approximately*** *14 months* ***old and evaluated samples (N=1,408) for*** *34 enteric viruses, bacteria, and parasites using qPCR (details in S2 Appendix).”*

29. 4º In the case of the environmental variable: only natural or artificial water bodies were recorded (for example, ponds in parks, drains or containment pools).

Response: Any surface water was included in our evaluation, including man-made and natural water bodies. We have updated the text to clarify this point.

Lines 338-341: “*Variables included: 1) seasonal surface water consistently present in a season, 2) ephemeral surface water present intermittently, and 3) any surface water (ephemeral, seasonal, or permanent) detected in the month of outcome measurement,* ***including man-made and natural water bodies****.”*

30. Proximity to sources of garbage accumulation could have been another interesting environmental variable to take into account and open dumps can be recorded through spectral signatures as well.

Response: We agree with the reviewer that this is an interesting avenue for future research. Unfortunately, it falls outside the scope of our current manuscript, which we attempted to keep focused on hydrometeorological risk factors.

31. Line 212: “We assessed effect modification for the diarrhea outcome by child age”. Why?

Response: We assessed effect modification by age because age is strongly associated with diarrhea risk. Therefore, it is useful to evaluate the difference across groups as children of different ages may be targeted for different interventions. We have added clarifying details and references to the text.

Lines 394-395: “*We assessed effect modification for the diarrhea outcome by child age (<1.5 vs. ≥1.5 years)* ***because age is strongly associated with diarrhea risk (49,50)****.”*

**Results**
Reviewer #1:

32. The results are clearly presented and the figures are effective.

Response: We are glad the reviewer found our results clearly and effectively presented.

33. Lines 192-195: In your pre-specified analysis plan, you state that “We will pool across all intervention arms from the original study to maximize statistical power and will include study arm as a covariate to adjust for any effects of study interventions on each outcome, except for the diarrhea outcome where we will conduct subgroup analyses separately within the control arm or intervention arms (all grouped together) due to the significant influence of the interventions on diarrhea in a seasonally-dependent manner.” However, in the manuscript, it seems like you exclude diarrhea data for the intervention arms. Please describe if and why this was changed.

Response: The reviewer is correct that we exclude data from the intervention arms for the diarrhea analysis. Early in our analysis, we noted a seasonally-dependent influence of the interventions on diarrhea and separated this analysis out into a parallel manuscript to present the interaction of interventions and weather risk factors on diarrhea. Thus, for this manuscript we restricted our analysis of the diarrhea outcome to observations collected at the study baseline (before intervention delivery) or the control arms of the study (for later time points), so this abrogated the need to do subgroup analyses. Reviewer #2 also raised a similar confusion, so we have moved the following text up to the Outcomes portion of the Methods section alongside where we describe the diarrhea measurements (it was previously in the Statistical Methods section where we believe it was not properly contextualized alongside the outcome descriptions):

Line 226-229: ***“The diarrhea cohort was restricted to the control arms of the original trial or data collected before intervention delivery (i.e. intervention arms at the baseline survey) (N=7,320 measurements) because intervention effects on diarrhea are modified by weather and environment (34).”***

We have also included this modification to the list of deviations from the pre-analysis plan in S1 Appendix:

5)	***“We pre-specified that we would pool across all intervention arms from the original study to maximize statistical power and include subgroup analyses within the control arm or intervention arms (all pooled together). In a parallel analysis, we observed a significant influence of intervention effectiveness on diarrhea in a seasonally-dependent manner, thus we restricted our analysis of the diarrhea outcome to only control arms, or outcomes collected at baseline before interventions were delivered. This abrogated the need to do subgroup analyses for the diarrhea outcome.”***

34. Line 337: Where are these results?

Response: We have added a supplemental figure to show the results of effect modification on all risk factors by child age (S11 Fig) and effect modification of temperature by precipitation (S2 Table). The text has been updated to read:

Lines 771-782:

*“****We detected meaningful effect modification in our pre-specified subgroup analyses evaluating child age category (6 months-1.5 years, 1.5-5 years) for the diarrhea outcome. Diarrhea in children aged 1.5-5 years old tended to be impacted by precipitation to a greater extent than younger children (S11 Fig). Heavy rain significantly increased diarrhea prevalence in older children (aPR 1.54, 95% CI 1.21, 1.97) but not those in the younger group (aPR 1.07, 95% CI 0.83, 1.37) in adjusted models with a 3-week lag (results were similar with a 2-week lag).***

***Due to the strong correlation between temperature and precipitation, we included models with both temperature and precipitation variables. Models which included both variables often had improved model fit (lower Akaike Information Criterion) over models with only temperature. However, there were few instances in which models were improved by the inclusion of an interaction term (S2 Table).****”*

35. S1 Appendix 2: I don’t see these additional results at the link included.

Response: We thank the reviewer for noting this. We had failed to make this subfolder publicly accessible but have corrected that.

Reviewer #2:

36. Line 221: Was the difference in diarrhea incidence in the rainy vs the dry season statistically significant?

Response: Yes, it was. We have added the prevalence ratio and 95% confidence interval to the text to clarify this.

Lines 454-456: *“Overall, diarrhea prevalence was 7.1%, with 6.1% prevalence in the dry season and 8.3% in the rainy season (Fig 1****B;*** ***prevalence ratio of rainy vs dry season 1.36, 95% CI 1.14, 1.62****).”*

37. Line 258: When you go from describing the distribution of the variables to reporting predictions of the model results, I would start a new paragraph and start it with “According to model results…” or something like that. If I’ve understood correctly, the prevalences reported in table S1 and lines 258 – 264 are predictions from GAMs at two values for temperature at either extreme of the range (and statistically significant results are in bold typeface in table S1). This is not clearly stated, however.

Response: The reviewer is correct that the prevalences reported are predictions from GAMs at two temperature values. We appreciate this suggestion to clarify that in the text and have edited the paragraph as suggested by moving the results to a separate paragraph starting with “According to model results…”.

Lines 488-499: *“During the study period, the average weekly temperature across the study area ranged from 15-33°C, the minimum weekly temperature ranged from 13-33°C, and the maximum weekly temperature ranged from 17-34°C. Enteropathogens were measured during a one-year period of the study, during which the minimum temperatures did not fall below 17°C and the average weekly temperature did not fall below 19°C.*

*According to adjusted model results using a 1-week lag, diarrhea prevalence was 3.3% (95% CI 2.0%, 5.6%) at average weekly temperature of 15°C and 6.8% (95% CI 5.2%, 8.9%) at 30°C; unadjusted models produced similar results (prevalence of 2.9% [95% CI 1.8%, 4.7%] at 15°C and 5.8% [95% CI 4.9%, 7.0%] at 30°C). Trends remained evident at longer lags, and the rate of increase in diarrhea prevalence was higher at weekly average temperatures above 30°C (Fig 3A). Higher average weekly temperatures were also associated with higher prevalence of Shiga toxin-producing E. coli….”*

38. I assume that rotavirus was excluded as a pathogen-specific outcome because it was detected in fewer than 10% of samples (same for astrovirus). Since rotavirus is a highly climate sensitive pathogen and readers will be looking for that result, I think that this should be stated somewhere (e.g., in the limitations paragraph).

Response: The reviewer correctly inferred that rotavirus did not meet our 10% threshold for pathogen-specific analysis as samples were collected from a community-based cohort, the majority of whom (86%) did not have diarrheal symptoms. We have added the following text to clarify the low prevalence of diarrhea in the methods and to make this specific point about rotavirus clear in the limitations paragraph.

Lines 255-259: *“****Stool samples were collected as part of a routine household visit regardless of children’s symptom status (14% of children reported diarrheal symptoms in the previous 7 days at the time of stool collection). We chose to collect stool from both asymptomatic and symptomatic children because prior literature has shown the importance of asymptomatic pathogen carriage on growth faltering (2,32,33).****”*

Lines 893-897: *“****Rotavirus, a highly climate-sensitive pathogen, was not included in our pathogen-specific analysis because it did not meet our 10% prevalence threshold (although detection of rotavirus is accounted for in our composite “Any virus” variable). Rotavirus was detected in only 2.2% of our community-based cohort, which is similar to other cross-sectional studies where the majority of samples are not associated with diarrheal symptoms.****”*

39. In line 285, an adjusted prevalence ratio (aPR) is reported, but after that, the abbreviation “PR” is used suggesting that the rest of the results are unadjusted. Similarly, in the coefficient plots, unadjusted PRs are plotted and the figure legends state that adjusted results are similar. I think it makes more sense to consistently report the adjusted results.

Response: We have modified the manuscript to report adjusted prevalence ratios and updated all plots/tables accordingly, including supplemental materials. In addition, we have added clarification to the methods to describe how the results for adjusted models with continuous exposures (e.g. temperature) are presented.

Lines 389-392: *“****We predicted prevalence from adjusted models with continuous exposures set at the median across all samples, intervention set at receiving both WASH and nutrition interventions, household wealth set at the lowest quartile, antibiotic use set at none in the previous 7 days, and sex set as male.”***

40. What possible causal explanation could there be for why aEPEC has a negative association with rainfall, but tEPEC has a positive one? I strongly suspect that this is the result of confounding. aEPEC, tEPEC and STEC are all diagnosed based on the presence of the highly prevalent eae gene and differentiated based on the combination of bfpA, stx1 and stx2 genes. That means that being positive for aEPEC, is not independent from being diagnosed with tEPEC or STEC. If a sample is positive for tEPEC or STEC, then by definition it is negative for aEPEC. Could it be that the strong positive effect of rainfall on tEPEC and STEC is making it appear as if there is a negative effect on aEPEC, when really, it’s that rainfall is making the EPEC more likely to be typical (or shiga-toxin producing) than atypical? I recommend excluding aEPEC or combining it with tEPEC into a single EPEC variable. Something similar could be going on with LT- and ST-ETEC.

Response: The reviewer makes an excellent point regarding confounding between the *E. coli* pathotypes for EPEC (aEPEC, tEPEC) and ETEC (ST-ETEC, LT-ETEC). We have combined the aEPEC and tEPEC outcomes into a single “EPEC” outcome and similarly combined ST-ETEC and LT-ETEC into “ETEC” in the revised manuscript and no longer report the individual types. With this new definition, the results for ETEC did not change, suggesting the main driver of the effect was from ST-ETEC. Results for the combined EPEC variable with respect to temperature and precipitation were null, suggesting there was potentially some confounding between the aEPEC and tEPEC variables as the reviewer had suggested.

41. Line 326: Include EAEC in the outcomes that decreased with above median surface water.

Response: Per another recommendation to present the adjusted results for all outcomes, we have updated this section and EAEC does not decrease under an adjusted model so we have not included it.

Reviewer #3:

42. The results of the participant and sample characteristics detailed in Table 1 are clear. However, the methodology regarding characteristics and sampling (e.g. time of year) is not equally clear in the text (without the need to refer to the Appendix).

We thank the reviewer for this feedback. In the revised manuscript, we have broken the diarrhea and enteropathogen outcomes into separate paragraphs and incorporated clarifying details from the appendix into the main manuscript text clarifying which children are included in each analysis and when the data was collected. The new sections read as follows:

Lines 224-259: *“****Diarrhea.*** *Field workers measured caregiver-reported diarrhea* ***in the previous 7 days*** *(three or more loose or watery stools in a 24-hour period or a single stool with blood) in children 6 months - 5.5 years old (N=4,478 children).* ***The diarrhea cohort was restricted to the control arms of the original trial or data collected before intervention delivery (i.e. intervention arms at the baseline survey) (N=7,320 measurements) because intervention effects on diarrhea are modified by weather (34).*** *Diarrhea prevalence was measured in three* ***main*** *rounds* ***from the diarrhea cohort*** ***(N = 6,589 observations; May 2012 - July 2013, September 2013 - September 2014, December 2014 - October 2015). This included index children (live births of women pregnant at the time of enrollment), other children living within the same compound that were younger than 3 years at study enrollment, and children within the same household born after the index children.*** ***Data on caregiver-reported diarrhea was collected in three additional rounds from children in the enteropathogen cohort (N = 731 observations from index children in the control arm; December 2012 - January 2014, November 2013 - November 2014, and March 2015 - March 2016).***

***Enteropathogens. The enteropathogen cohort included index children from a subsample of clusters that was evenly balanced across the control, WSH, nutrition, and N+WSH arms (allocation ratio 1:1:1:1). Clusters in each arm were selected based on logistical feasibility for specimen collection and transport to a central laboratory.*** *Field workers collected stool samples from* ***these*** *children* ***between November 2013 and November 2014 when they were approximately 14 months old and evaluated samples (N=1,408) for*** *34 enteric viruses, bacteria and parasites using qPCR (details in S2 Appendix)****. Stool samples were collected as part of a routine household visit regardless of children’s symptom status (14% of children reported diarrheal symptoms in the previous 7 days at the time of stool collection). We chose to collect stool from both asymptomatic and symptomatic children because prior literature has shown the importance of asymptomatic pathogen carriage on growth faltering (2,32,33).****”*

43. Line 232. “The study period spanned three rainy seasons (empirically determined as the period when the 5-day rolling average rainfall was ≥10mm/day) from 2013-2015”: this information should be moved to Methodology

Response: We have moved the definition of the rainy season to the methods section, as the reviewer has recommended. It can be found at the end of the section where we describe the precipitation data. We have also added justification for the use of this method, per another reviewer’s recommendation.

Lines 330-335: *“****Although prior studies have used different fixed periods between the months of June and October as the rainy season in Bangladesh (30,41,42), we observed variations in the start and end of the rainy period in different years of our study, with substantial rainfall before June in every year.*** ***Therefore****, we determined the rainy season empirically* ***(10),*** *defined as the continuous period during which the 5-day rolling average of daily precipitation was ≥10mm/day.”*

**Conclusions**

Reviewer #1:

44. Line 421-430: Others have found that strict adherence to WASH interventions is necessary to realize public health gains (<https://journals.plos.org/plosone/article?id=10.1371/journal.pone.0036735>). It might be worth acknowledging this evidence in your recommendation. How does this impact your conclusion?

Response: We agree that adherence is an important aspect of the efficacy of WASH interventions. We have added the following text to the Discussion, which includes the reference kindly provided by the reviewer (reference 57):

Lines 917-921: *“Of note, the enteropathogens that had higher prevalence under higher temperature and precipitation levels are not currently vaccine-preventable.* ***Many can be prevented by household WASH interventions (51), but sustained use is critical to realizing health benefits (58,59) and has proven difficult to maintain in the long term (60–62).****”*

45. Line 421-430: I think this is a valid recommendation, but it is oversimplified and “targeting” doesn’t really distinguish between promotion and use. Are you suggesting that households should only treat their water in the rainy season? I assume not. Others have hypothesized that very frequent and intensive promotion is necessary to achieve high adherence. You could make your recommendation more specific and thus more meaningful. E.g., recommend that implementers consider incorporating annual promotion (or increasing the frequency of their promotion) during the rainy season (you might come to a different conclusion, this is just an example).

Response: We appreciate the nuance of the reviewer’s thinking with respect to targeting of interventions. We support policies that would increase promotion and/or provide direct resources to households to offset the cost of disinfectant/soap to maximize adherence to water and handwashing interventions. We have added the following text to the Discussion:

Lines 921-923: *“Given the high cost and user burden of* ***household*** *WASH interventions (60), seasonal targeting* ***via the direct provision of resources and/or increased promotion*** *may improve both public health impact and cost-effectiveness.”*

46. I am curious if you have a hypothesis for why virus trends are non-linear with temp (Fig 3)? It’s interesting...

Response: In the revised manuscript, we present adjusted analyses, and the only viruses displaying non-linear trends are norovirus and any virus (with a pattern strongly mirroring the most prevalent virus, norovirus). While the pattern for norovirus across temperature is somewhat nonlinear, for temperatures <30 C, the confidence interval is wide enough that the true pattern could be linear within that range. Thus, we are hesitant to over-interpret the shape of that relationship. The pattern for any virus appears to be a bit more nonlinear because it captures the differing patterns with temperature for norovirus and sapovirus.

47. You might include more discussion of potential mechanisms for the relationships between weather, infection, and diarrhea in the discussion and/or the introduction. Could behavioral factors play a role? There may be some relevant discussion here: <https://www.sciencedirect.com/science/article/pii/S0048969721069527#s0105> and here:
    <https://pubs.acs.org/doi/full/10.1021/acs.est.2c07284> I am also curious about your thoughts on mechanisms that are pathogen-specific.

Response: We have added additional detail and references to the introduction

Lines 160-174: “*Increased temperatures have been associated with higher incidence of bacterial diarrhea but lower incidence of viral diarrhea (16,****18****).* ***Higher temperatures are also associated with less Escherichia coli on children’s hands, more cases of food-borne salmonellosis, and higher E. coli concentrations on food, in source water, and in stored drinking water in the household (19–21). Higher relative humidity can increase the viability of bacteria but increase viral inactivation, while also increasing the transfer efficiency of both from fomites (22–24).*** *In LMICs, higher temperatures have been associated with a decrease in rotavirus prevalence and small increase in adenovirus prevalence; higher humidity and temperature have been associated with higher enteric bacteria prevalence and lower virus prevalence; and associations with precipitation varied by pathogen (9).* ***Furthermore, human behaviors such as handwashing and water treatment practices have been shown to vary under differing weather conditions. For example people are less likely to wash their hands but more likely to boil their drinking water during colder weather, and the use of water storage and treatment has been shown to increase in dry periods (25).”***

48. Lines 358-360: You might mention the concentration-dilution hypothesis (and other potential mechanisms) in the intro.

Response: We have added the concentration-dilution hypothesis explicitly to the introduction.

Lines 153-159: *“Prior studies have found increased diarrhea after heavy rainfall and floods****, with at least some attribution given to the flow of fecal material into the environment from overwhelmed sewage treatment facilities in high-income contexts and latrine overflow/leakage in low- and middle-income contexts*** *(13–15).* ***P****arasitic and bacterial diarrhea are more common in the rainy season (12,16).* ***The concentration-dilution hypothesis posits that the greatest risk will occur at the beginning of a rainy season, after an antecedent period of no rainfall where pathogens can accumulate in the environment. Data have supported this hypothesis in most contexts (17).****”*

49. Line 383-387: Hmm. Perhaps there is runoff/pathogen transport for some period of time after a heavy rainfall event, thus taking some time to reach participants, their water sources, their food etc? Or people store their water?

Response: We consider it unlikely that water storage is responsible for these results as water is typically not stored for more than a day in this setting. We have amended the discussion to include these other potential mechanisms.

Lines 855-863: “*Particularly for bacteria, analyses using 2- and 3-week lag periods had stronger associations than those with a 1-week lag; this may reflect higher* ***environmental growth, survival, and/or delayed transport (e.g. from flooding that occurs days after a rain event) following higher precipitation****.* ***Adenovirus 40/41, a double-stranded DNA (dsDNA) virus, displayed an inverse relationship with precipitation compared to the other viruses evaluated, which were both single-stranded RNA (ssRNA) viruses. A prior study showed that ultraviolet irradiation led to a 4-log10 inactivation of ssRNA viruses but only a 2-log10 inactivation of dsDNA viruses in water (53). It is possible that increased water turbidity associated with precipitation events intensifies this relationship.****”*

Reviewer #2:

50. Here are some of my key takeaways from this study, which I think deserve more emphasis. The effects of precipitation on all-cause diarrhea were modest and not statistically significant. This may be because treating this catch-all diagnostic category as a single outcome masks heterogenous effects on distinct pathogen taxa (bacteria, viruses, parasites) and species. Associations between precipitation and bacteria were almost all direct (especially if you discount aEPEC for reasons mentioned above). The null findings for any parasites mask a strong direct effect of precipitation on Cryptosporidium across increasing lag lengths, that is offset by a protective effect on Giardia. Associations with viruses were inverse with one exception. Adenovirus prevalence increased with above average rainfall but decreased with above average surface water (contrast with <https://doi.org/10.3390/ijerph17020487>). The authors rightly engage with the concentration-dilution hypothesis and are transparent about the study’s limitations in assessing this. According to the concentration-dilution hypothesis, the effect of rainfall on (all-cause) diarrhea is non-linear, most pronounced at the extremes, and modified by antecedent saturation conditions (Kraay et al. 2020). I think you could make a case from the present study’s findings that, by focusing on broad diarrheal outcomes, the hypothesis oversimplifies things, or at least that, if true, it is mediated by differential impacts on different pathogen taxa and species at either precipitation extreme.

Response: We appreciate the reviewer’s thoughtful comment and have included more emphasis on the heterogeneity of effects across different enteropathogens in the results and discussion.

Lines 114-116 (Abstract): *“****Our findings emphasize the heterogeneity of the relationships between hydrometeorological variables and specific enteropathogens, which can be masked when looking at composite measures like all-cause diarrhea.****”*

Lines 574-576: *“****Associations between temperature and the combined metrics of ‘any virus’ or ‘any parasite’ masked the heterogeneous effects of individual viral or parasitic enteropathogens (Fig 3D and 3E).”***

Lines 794-798: *“****Consistent with prior studies, our analysis found that the effects between hydrometeorological variables and enteropathogens are heterogeneous (9,14,16). We emphasize the heterogeneity even within pathogen class, such that effects can be masked when looking at composite variables (e.g. any viral enteropathogen) or even at the level of all-cause diarrhea.”***

Lines 823-826: *“Taken together, our findings suggest that in our study setting, even moderately higher precipitation was associated with meaningful increases in diarrhea.* ***However, these results obscure important heterogeneous impacts of precipitation on specific enteropathogens, which we discuss below.****”*

Lines 858-863: *“****Adenovirus 40/41, a double-stranded DNA (dsDNA) virus, displayed an inverse relationship with precipitation compared to the other viruses evaluated, which were both single-stranded RNA (ssRNA) viruses. A prior study showed that ultraviolet irradiation led to a 4-log10 inactivation of ssRNA viruses but only a 2-log10 inactivation of dsDNA viruses in water (53). It is possible that increased water turbidity associated with precipitation events intensifies this relationship.”***

Lines 876-880: *“Our findings reinforce that relationships between temperature and enteropathogen carriage are heterogeneous,* ***even within pathogen class (i.e. virus, bacteria, parasite).*** ***The null results for the composite measures of ‘any virus’ or ‘any parasite’ in the background of significant effects for individual viruses/parasites cautions against evaluating higher-level groupings that may mask pathogen-specific effects.****”*

51. Limitations and policy implications are nicely enumerated.

Response: We are happy that the reviewer found our discussion of limitations and policy implications well-reasoned.

Reviewer #3:

52. Avoid very long sentences: Line 344. Finish a sentence and start a new one. “ …in rural Bangladesh, we found… “ replaced by “…in rural Bangladesh. We found…”

Response: We appreciate the reviewer’s overall comment regarding using shorter sentences to improve clarity. We have strived to break long sentences into two separate sentences throughout the manuscript in this revision. In this instance, the meaning of the sentence is changed if edited as suggested, so we have decided to keep the sentence intact.

53. Line 346. Finish a sentence and start a new one. “ … pathogens, and surface water
    … “ replaced by “…pathogens. Surface water…”.

Response: We have edited the text as suggested.

54. Line 347. “Our findings suggest that under projected increases in temperature (mean increase of 1° C) and precipitation (mean increase of 6%) from 2021-2050 in Bangladesh due to climate change (33), there would be small increases in the prevalence of diarrhea and certain enteric pathogen infections.” This sentence is very long and I do not think it is relevant in this paragraph where the main findings are summarized.

Response: We have edited this text by breaking it into 2 separate sentences. We have also moved this text to later in the discussion under a new subheading titled Climate and policy implications.

Lines 904-911: *“Climate change models predict increases in temperature (mean increase of 1° C) and precipitation (mean increase of 6%) in Bangladesh from 2021-2050 (55). Our findings suggest that under these projections, there would be small increases in the prevalence of diarrhea and certain enteric pathogen infections.”*

**Editorial and Data Presentation Modifications** 
Reviewer #1:

55. Line 62: Why use % for temp results and 1.25 fold for precipitation? I think that 25% would mean the same thing and be more clear.

Response: We have updated the abstract to report precipitation results as a percentage to be consistent with the temperature results. The updated text reads:

Lines 62-64: *“Above-median weekly precipitation (median: 13mm; range: 0-396mm) was associated with* ***29% higher*** *diarrhea (adjusted prevalence ratio 1.29, 95% CI 1.07, 1.55)….”*

56. Line 284: ”Above median” weekly precip = above overall median (not median for that season). At first I thought you were saying you used the seasonal median for your variable definition based on the way these sentences are ordered. Recommend adding “(13mm)” after “above-median” for clarity.

Response: The reviewer has correctly understood that the above median weekly precipitation sum is based on the overall median, not a season-specific median. As recommended, we have edited the text here (and in other places where we discuss the above-median results) to include the median cutoff in parenthesis so the cutoff value is clear to readers. We have also added the following clarifying statement in the methods section.

Lines 313-326 (Methods): *“We created an indicator for heavy rainfall (any day in the prior week with total daily precipitation >80th percentile* ***[17mm]*** *on rainy days during the study period) and an indicator for whether the weekly sum of precipitation (over the past 7 days) was above or below the median* ***(13mm)*** *for the entire study period.* ***Precipitation thresholds were based on the percentile of all daily totals (across all study years and not season-specific) in order to maximize the generalizability of our results.****”*

Line 599: *“Above-median weekly total precipitation* ***(>13mm)*** *was associated…”*

57. Line 385: Typo, should this read “Particularly for bacteria”?

Response: We appreciate the reviewer catching this typo. We have corrected the text as suggested to “Particularly **for** bacteria, …”

Reviewer #2:

58. Consider using the word “hydrometeorological” as a catch-all term for your main exposures.

Response: We thank the reviewer for this suggestion and have modified the text throughout to use the term hydrometeorological to describe our exposure variables. Examples include:

Manuscript title: *“Influence of* ***hydrometeorological*** *risk factors on child diarrhea and enteropathogens in rural Bangladesh”*

Lines 189-190: “*We hypothesize that there are pathogen-specific differences in the direction, magnitude , and significance of associations with* ***hydrometeorological*** *exposures.”*

Lines 271-274: [This includes changing the section heading in the methods section describing the exposure variables]:

***“Hydrometeorological*** *variables*

*We matched* ***hydrometeorological*** *risk factors from remote sensing datasets to trial data by date of outcome measurements and geocoordinates of study compounds (groups of households in which patrilineal families share a common courtyard).”*

59. Figure 1: The star presumably represents Dhaka, but is not labelled. State months of the season. Give the panels letters (as in the other figures) rather than referring to them by left and right. There are many compounds in the right panel that aren’t in the left. If the lower scale really does include the value 0, then they should show up as yellow. Was follow-up different in the two seasons?

Response: We have added text to the figure label indicating that the blue star represents Dhaka and added panel letters. We used a data-based definition of season in order to account for temporal heterogeneity in rainfall between years; thus, we did not list specific months for each season as they varied between years (see reviewer comment #6 above for details).

The reviewer correctly noted that there are many compounds only visited during a single season. This is due to how the diarrhea data was collected. To optimize field data collection, compounds were visited in a geographically ordered way such that nearby villages were visited around the same time before the study team moved to another geographic area. Each annual visit started in the same place and progressed along the same geographic path, thus compounds which were visited during the rainy season in the first year are more likely to be visited in the rainy season in subsequent years. We have incorporated this text into the Outcomes section of the methods to help clarify this point to our readers.

Lines 206-212: “***Approximately 54 clusters were sampled per month from the diarrhea cohort, and 14 clusters were sampled per month from the enteropathogen cohort in a spatially dependent manner as the study team moved around the study area over the course of a year. Each round of household visits started in the same place and progressed along the same geographic path, thus compounds which were visited during the rainy season in the first year were more likely to be visited in the rainy season in subsequent years.****”*

60. Figure 2: “For periods when there was no diarrhea data collection, we display the risk factor values for the next biweekly period with at least 10 diarrhea measurements.” What is the reason for doing this? Why not plot a continuous time series of the weather variables in panels B - D, since whether or not diarrhea was recorded in a given week has no bearing on them.

Response: The reviewer makes a good point. However, the way in which we are presenting the data includes the calculation of 95% confidence intervals using robust sandwich standard errors to account for correlation within study clusters. For temperature and precipitation data, the data had daily resolution and we were able to capture the data for all pixels within our study area, so we were able to obtain community-wide averages for the duration of the study period. However, the surface water and humidity raw data had only monthly resolution and the raw data was processed in such a way that it was matched to households GPS coordinates immediately and we did not capture the information for all households within the community (e.g. we do not have an estimate of the distance to any surface water for every pixel in the study area for every month of the entire study period, only for the specific GPS coordinates of households included in our study and the month of outcome data collection). The fact that we didn’t collect these data for all time periods prevents us from presenting the data for the time periods corresponding to the missing diarrhea data. We also decided that presenting these variables also with 95% confidence intervals using robust standard errors was most appropriate to account for the clustered study design since the data was only obtained from these specific households. As the reviewer points out, we could present the temperature and precipitation data for weeks without diarrhea outcome measurement since we do have those, but we have opted not to so that our plot is consistent across all facets.

61. Figure 3: Panel C (bacteria) has too many lines to discern any individual pathogen’s pattern. Consider excluding less pathogenic bacteria (Aeromonas, Plesiomonas) or having a separate panel for E. coli pathotypes.

Response: We have broken pathogenic *E. coli* out into their own panel to facilitate easier discernment of individual pathogen patterns, and removed *Plesiomonas* from the main bacteria plot to facilitate discerning the other data.

62. Figure 4: State that the axis is on the log scale.

Response: We have added this clarification to the figure legend (and also to legends in supplementary figures).

Line 686: *“****The x-axis is on the log scale.****”*

Reviewer #3:

63. Please, avoid long sentences.
    Some editing suggestions were added in the different sections.

Response: We appreciate the reviewer’s overall comment regarding using shorter sentences to improve clarity. We have strived to break long sentences into two separate sentences throughout the revised manuscript. We have addressed the specific edits suggested by the reviewer in comments #52, #53, & #54.

**Summary and General Comments**
Reviewer #1:

64. This is an important paper that should be published. Linking weather to specific pathogens is a valuable and relatively novel contribution in an area that is currently under-researched. Empirical data such as the data provided in this paper is necessary for understanding potential climate change impacts and improving the accuracy of predictive models. The authors present their work clearly and the methods are sound.

    Please consider including the following additional analyses, or describe why they are not needed:
    - The effects of temperature and precipitation in combined models, or address this further and provide a justification for not doing so.

Response: In the updated manuscript, we include temperature and precipitation in a combined model, as well as models that include an interaction term. We have included the following text in the Methods and Results sections, respectively:

Lines 395-444: *“****Because pair-wise correlations between temperature and precipitation were strong (S1 Fig), we evaluated temperature exposure models with and without inclusion of precipitation as an additional covariate. We also assessed effect modification of temperature by precipitation using a model with both exposures and an interaction term****.”*

Lines 778-782*: “****Due to the strong correlation between temperature and precipitation, we included models with both temperature and precipitation variables. Models which included both variables often had improved model fit (lower Akaike Information Criterion) over models with only temperature. However, there were few instances in which models were improved by the inclusion of an interaction term (S2 Table).***”

65. If you do include temp and precip in combined models, you might also consider testing whether the inclusion of an interaction term between temp and precip improves the model.

Response: See above responses to #64.

Reviewer #2:

66. Congratulations on a fascinating and well executed analysis that is mostly well written and with appealing and interpretable data visualizations. As the authors convincingly point out, given the exacerbating effects of climate change on diarrheal disease incidence and lingering uncertainties such as the concentration-dilution hypothesis, it is important to tease out the effects of specific environmental parameters on individual pathogen etiologies and the pathways through which these associations operate. The authors make full use of a unique and opportune dataset to address this. The literature cited is relevant and up to date (though here’s one that may have come out since submission 10.1001/jamapediatrics.2023.3964), the analytical methods are sound, and the use of the surface water variables is novel.

Response: We thank the reviewer for pointing out new relevant literature and have added the suggested manuscript to our introduction where we discuss increased diarrhea after floods (Lines 153-156).

Reviewer #3:

67. Abstract. It would be good to describe the study design used in this work.

Response: We have included in the abstract that the data was collected longitudinally during 2012-2016.

Lines 53-55: “*We matched temperature, precipitation, surface water, and humidity data to observational* ***longitudinal*** *data from a cluster-randomized trial that measured diarrhea and enteropathogen prevalence in children 6 months-5.5 years from 2012-2016.”*

68. Introduction. The introduction is well presented, addressing the issue of diarrhea and the influence of climatic and environmental factors. The general objective is well stated. However, it would be appropriate to incorporate some prediction of what they would expect to find considering the prevalence of diarrhea and enteroparasites and the climatic and environmental characteristics of the country.

Response: We have included the following hypothesis at the end of the introduction:

Lines 189-190: *“****We hypothesize that there are pathogen-specific differences in the direction, magnitude, and significance of associations with hydrometeorological exposures.****”*
